# Supplementary material for: Study protocol for a single-blind, parallel-group, randomised, controlled non-inferiority trial of 4-day intensive versus standard cognitive behavioural therapy for adults with obsessive–compulsive disorder
Source: BMJ Open. 2023 Dec 14;13(12):e076361. doi: 10.1136/bmjopen-2023-076361 (PMC10729159; doi:10.1136/bmjopen-2023-076361)
Supplement: Supplementary data [file bmjopen-2023-076361supp001.pdf]

Study protocol for a single-blind, parallel-group, randomized, controlled non-inferiority trial of four-day intensive versus standard cognitive behavioral therapy for adults with obsessive-compulsive disorder

Supplemental table 1. Overview of the recruitment, treatments and assessment points.

	Enrollment	Allocation	Pre-treatment	Week 1-2	Week 4	Week 5-6	Week 7	Week 8-14	Week 15 (primary end-point)	7-month follow-up	16-month follow-up
Enrollment:											
Eligibility screen	X										
Informed consent	X										
Allocation		X									
Treatment											
Gold-standard CBT				X	X	X	X	X			
B4DT				X							
Clinician-rated instruments											
SCID-5 (OCD)	X										
MINI	X										
Y-BOCS	X				X		X		X	X	X
CGI-S	X				X		X		X	X	X
CGI-I					X		X		X	X	X
PEAS*					X				X		
Self-rated instruments											
YBOCS-SR			X						X	X	X
OCI-R			X						X	X	X
MADRS-S			X						X	X	X
WSAS			X						X	X	X
AQoL-6D			X						X	X	X
TIC-P			X						X	X	X
NEQ									X	X	X

Other adverse events					X		X		X	X	X
CEQ**				X							
WAI-SR**				X							
Treatment preference			X						X		

Abbreviations: AQoL-6D, Assessing Quality of Life 6 Dimensions; CEQ, Credibility/Expectancy Questionnaire; CGI-I, Clinical Global Impression–Improvement; CGI-S, Clinical Global Impression–Severity; MADRS-S; Montgomery-Åsberg Depression Rating Scale–Self-Rated; MINI, Mini International Neuropsychiatric Interview; NEQ, Negative Effects Questionnaire; OCI-R, Obsessive-Compulsive Inventory–Revised; PEAS, Patient Exposure/Response Prevention Adherence Scale; SCID-5, Structured Clinical Interview for DSM-5 Disorders; TIC-P, Treatment Inventory of Costs in Psychiatric Patients; WAI-SR, Working Alliance Inventory–Short Form Revised; WSAS, Work and Social Adjustment Scale; Y-BOCS, Yale-Brown Obsessive Compulsive Scale; Y-BOCS-SR, Yale-Brown Obsessive Compulsive Scale–Self-Rated; Other adverse events, semi-structured questions about potential severe adverse events and psychiatric care outside the study

* PEAS rated once at the last day of B4DT treatment in the B4DT-arm

** CEQ and WAI-SR rated by participants at the last day of B4DT treatment and week 2 in individual CBT
