# Supplementary material for: Study protocol for a single-blind, parallel-group, randomised, controlled non-inferiority trial of 4-day intensive versus standard cognitive behavioural therapy for adults with obsessive–compulsive disorder
Source: BMJ Open. 2023 Dec 14;13(12):e076361. doi: 10.1136/bmjopen-2023-076361 (PMC10729159; doi:10.1136/bmjopen-2023-076361)
Supplement: Author's manuscript [file bmjopen-2023-076361.draft_revisions.pdf]

BMJ Open is committed to open peer review. As part of this commitment we make the peer review history of every article we publish publicly available.

When an article is published we post the peer reviewers' comments and the authors' responses online. We also post the versions of the paper that were used during peer review. These are the versions that the peer review comments apply to.

The versions of the paper that follow are the versions that were submitted during the peer review process. They are not the versions of record or the final published versions. They should not be cited or distributed as the published version of this manuscript.

BMJ Open is an open access journal and the full, final, typeset and author-corrected version of record of the manuscript is available on our site with no access controls, subscription charges or pay-per-view fees (http://bmjopen.bmj.com).

If you have any questions on BMJ Open's open peer review process please email info.bmjopen@bmj.com

BMJ Open

Study protocol for a single-blind, parallel-group, randomized, controlled non-inferiority trial of four-day intensive versus standard cognitive behavioral therapy for adults with obsessive-compulsive disorder

Journal:	*BMJ Open*
Manuscript ID	bmjopen-2023-076361
Article Type:	Protocol
Date Submitted by the Author:	05-Jun-2023
Complete List of Authors:	Ivanova, Ekaterina; Karolinska Institutet, Center for Psychiatry Research, Department of Clinical Neuroscience; Region Stockholm, Stockholm Health Care Services<br>Fondberg, Robin; Karolinska Institutet, Centre for Psychiatry Research, Department of Clinical Neuroscience<br>Flygare, Oskar; Karolinska Institutet, Center for Psychiatry Research, Department for Clinical Neuroscience; Region Stockholm, Stockholm Health Care Services<br>Sannemalm, Max; Karolinska Institutet, Centre for Psychiatry Research, Department of Clinical Neuroscience; Region Stockholm, Stockholm Health Care Services<br>Asplund, Sofia; Region Stockholm, Stockholm Health Care Services<br>Dahlén, Sofia; Region Stockholm, Stockholm Health Care Services<br>Sampaio, Filipa; Uppsala Universitet, Department of Public Health and Caring Sciences<br>Andersson, Erik; Karolinska Institutet, Division of Psychology, Department of Clinical Neuroscience<br>Mataix-Cols, David; Karolinska Institutet, Centre for Psychiatry Research, Department of Clinical Neuroscience; Region Stockholm, Stockholm Health Care Services<br>Ivanov, Volen; Karolinska Institutet, Centre for Psychiatry Research, Department of Clinical Neuroscience; Region Stockholm, Stockholm Health Care Services<br>Rück, Christian; Karolinska Institutet, Centre for Psychiatry Research, Department of Clinical Neuroscience; Region Stockholm, Stockholm Health Care Services
Keywords:	Adult psychiatry < PSYCHIATRY, Anxiety disorders < PSYCHIATRY, PSYCHIATRY

SCHOLARONE™
Manuscripts

Study protocol for a single-blind, parallel-group, randomized, controlled non-inferiority trial of four-day intensive versus standard cognitive behavioral therapy for adults with obsessive-compulsive disorder

Document date: 05/6/2023
Document version: 1.1
Word count: 3,907

Authors: Ekaterina Ivanova[1]*, Robin Fondberg[2]*, Oskar Flygare[1], Max Sannemalm[1], Sofia Asplund[3], Sofia Dahlén[3], Filipa Sampaio[4], Erik Andersson[5], David Mataix-Cols[1,6], Volen Ivanov[1], Christian Rück[1]

**=contributed equally*

Author affiliations:

[1] Centre for Psychiatry Research, Department of Clinical Neuroscience, Karolinska Institutet, & Stockholm Health Care Services, Region Stockholm, Sweden

[2] Centre for Psychiatry Research, Department of Clinical Neuroscience, Karolinska Institutet, Sweden

[3] Stockholm Health Care Services, Region Stockholm, Sweden

[4] Department of Public Health and Caring Sciences, Uppsala University, Uppsala, Sweden

[5] Division of Psychology, Department of Clinical Neuroscience, Karolinska Institutet, Sweden

[6] Department of Clinical Sciences, Lund University, Lund, Sweden

Corresponding author: Ekaterina Ivanova, ekaterina.ivanova@ki.se, Huddinge sjukhusområde M46, Medicingatan 6, plan 4, 141 86 STOCKHOLM

For peer review only - http://bmjopen.bmj.com/site/about/guidelines.xhtml

ARTICLE SUMMARY

Abstract

Introduction

Individual cognitive behavioral therapy (CBT) with exposure and response prevention (ERP) is an effective treatment for obsessive-compulsive disorder (OCD). However, individual CBT is costly and time-consuming, requiring weekly therapy sessions for 3 to 4 months. A 4-day intensive version of CBT for OCD delivered in group format has been recently developed in Norway (Bergen 4-Day Treatment, B4DT). B4DT has shown promising results in several uncontrolled and one small, randomized trial, but its non-inferiority to the gold-standard treatment has not been established.

Methods and analysis

This single-blind, randomized controlled trial including 120 patients (60 per arm) will compare B4DT to individual CBT. The primary outcome is the blind assessor-rated Yale-Brown Obsessive-Compulsive Scale (Y-BOCS). We hypothesize that B4DT will be non-inferior to gold standard CBT 15 weeks after treatment start. The non-inferiority margin is set at 4 points on the Y-BOCS. Secondary outcomes include time to treatment response, cost effectiveness, response and remission rates, dropout rates, and adverse events.

Ethics and dissemination

This study has been approved by the Swedish Ethical Review Authority. Hypotheses were specified and analysis code published before data collection started. Results from all analyses will be reported in accordance with the Consolidated Standards of Reporting Trials statement for non-pharmacological trials (CONSORT) and Consolidated Health Economic Evaluation Reporting Standards (CHEERS) irrespective of outcome.

Trial registration number

Clinicaltrials.gov; NCT05608278. Open Science Framework; https://osf.io/w5bfp/.

Keywords: Obsessive-compulsive disorder, cognitive behavior therapy, intensive treatment

For peer review only - http://bmjopen.bmj.com/site/about/guidelines.xhtml

Strengths and limitations

- If effective, B4DT could reduce treatment duration and provide an evidence-based alternative to standard CBT for patients with OCD
- High statistical power to test the primary hypothesis that B4DT is not more than 4 points worse on Y-BOCS than gold standard CBT 15 weeks after treatment start
- Prespecified methodology and analysis code
- Low statistical power to robustly evaluate the secondary outcomes
- Participants will receive treatment at clinics located in different parts of Stockholm, which may be an obstacle for recruitment and retention.

INTRODUCTION

Obsessive-compulsive disorder (OCD), is characterized by recurrent uncontrollable (obsessions), and repetitive behaviors (compulsions) (1), is highly disabling (2,3), has a lifetime prevalence of 2-3% (4) and is associated with increased morbidity and mortality (5,6), academic underachievement (7) and labor-market marginalization (8).

Cognitive behavioral therapy (CBT) with exposure and response prevention (ERP) is a well-established treatment for OCD (9,10), recommended by international guidelines as a first-line treatment for the disorder (11). CBT is most commonly delivered in an individual format with weekly sessions during three to four months (12). This means that patients experience a relatively slow road to recovery. If treatment could be delivered in a more intensive way, for a shorter period, without sacrificing clinical efficacy, patients could return to their normal lives earlier. Furthermore, this may potentially result in more cost-effective use of clinical resources.

Researchers in Norway have recently developed an intensive version of CBT for OCD - the Bergen 4-Day Treatment (B4DT) (13). Results from uncontrolled trials indicate that approximately 90% of patients reliably improved (14–17) and that 70% were in remission four years after treatment (16). A recent RCT (n=48) showed that B4DT was superior both to a self-help book for OCD and a wait list control (18). Thus, B4DT is potentially effective and attractive for patients and caregivers alike, but its efficacy and cost-effectiveness have not yet been investigated in a head-to-head comparison with gold-standard individual CBT.

In this single-blind, non-inferiority randomized controlled trial, we will evaluate the efficacy of B4DT compared to gold standard, individual CBT. If, as we hypothesize, B4DT is non-inferior to traditional CBT, it could expand the range of available evidence-based options for this patient group.

For peer review only - http://bmjopen.bmj.com/site/about/guidelines.xhtml

Aim and objectives

The primary objective of the study is to evaluate if the intensive Bergen 4-day treatment (B4DT) is a non-inferior alternative to gold standard individual cognitive behavioral therapy (CBT) for reducing symptom severity in adults with OCD.

We will compare the two groups 15 weeks after treatment start (primary endpoint) with regard to OCD severity. We hypothesize that B4DT will be non-inferior to standard CBT using a non-inferiority margin of 4 points on the Yale-Brown Obsessive-Compulsive Scale (Y-BOCS) (19).

Our secondary objectives are:

1) (Primary endpoint) To test whether B4DT is superior to standard CBT
2) (Primary endpoint) To compare B4DT and gold standard CBT with regards to:
    a. Speed of response
    b. Rates of response and remission
    c. Dropout rate
    d. Cost-effectiveness
    e. Adverse events

    Due to its short and intensive format, we expect B4DT to provide a faster response than gold-standard CBT. For secondary objective 2.b-2.e, we have no directed hypotheses. As this study is not sufficiently powered to make confirmatory claims based on the results of these secondary analyses, results will be interpreted as exploratory.

3) Long-term maintenance of gains

METHODS

Study Design

We will conduct a single-blind (blinded outcome assessors), randomized (1:1), controlled, parallel-group, non-inferiority trial comparing intensive 4-day CBT (B4DT) and gold standard individual CBT for adults with OCD residing in Sweden. A total of 120 participants will be assessed before treatment start, at week 4, 7, and 15 (primary endpoint), and 7- and 16 months after treatment start (follow-ups). The Consolidated Standards of Reporting Trials (CONSORT) flow chart of the trial is shown in Figure 1. The outcome measures, statistical models and cut-offs, time-points of evaluation, and analysis code were published on osf.io (https://osf.io/w5bfp/) before data collection began. The study will follow Good Clinical Practice (GCP) and all quality- and safety aspects will be regularly monitored by an external party, the Karolinska Trial Alliance. The study has been approved by the Swedish Ethical Review

For peer review only - http://bmjopen.bmj.com/site/about/guidelines.xhtml

For peer review only

Authority (EPM 2022-01713-01). Potential protocol modifications will be described in detail on osf.io, clinicaltrials.org, and – if the modification touches upon an ethical issue – an amendment will be submitted to the Swedish Ethical Review Authority.

[Figure 1 at about here]

Participants

Patients referred to one of the two involved OCD specialist clinics in Stockholm and self-referred patients will be assessed for eligibility by a staff clinician during a full psychiatric assessment. Candidates that live within approximately one hour of both clinics and agree to participate irrespective of allocation site will be offered inclusion if they match the inclusion/exclusion criteria (see Table 1). Informed consent will either be obtained during a face-to-face clinician visit or on an encrypted study platform, requiring identification and two-step authentication. Once the patient is included in the trial, data on any concurrent treatments will be collected, but those will not automatically lead to discontinuation of the trial by the patient. Karolinska trial alliance is planning a total of seven visits to the study sites, during which they will monitor presence of signed informed consent, documentation of inclusion/exclusion criteria and randomization for each participant.

Table 1. Overview of inclusion and exclusion criteria

**Inclusion criteria**	≥ 18 years of age.
	Primary diagnosis of OCD according to DSM-5.
	Clinician-rated Y-BOCS score of ≥ 16.
	Written informed consent.
	Being willing and able to attend treatment at any one of the two treatment clinics, regardless of the clinic where the initial assessment took place (the two clinics are located at different locations in Stockholm, about 20 km apart).
	Being fluent in Swedish.
**Exclusion criteria**	Other psychological treatment for OCD planned during the trial period.
	Completed CBT with exposure and response prevention (ERP) for OCD in the last 12 months.
	Changes in psychotropic medication within the last 2 months.
	Bipolar disorder.
	Psychosis.

For peer review only - http://bmjopen.bmj.com/site/about/guidelines.xhtml

	Alcohol or substance dependence.
	Organic brain disorder.
	Hoarding disorder or OCD with primary hoarding symptoms.
	Suicidal ideation that would warrant close monitoring.

Abbreviations: CBT, cognitive behavior therapy; DSM-5, The Diagnostic and Statistical Manual of Mental Disorders, Fifth Edition; OCD, obsessive-compulsive disorder; Y-BOCS, Yale-Brown Obsessive-Compulsive Scale.

For peer review only

Randomization and concealment

The included participants are randomized consecutively to receive either B4DT (delivered at Ångestenheten, Psykiatri Nordväst), or gold standard CBT (delivered at OCD-programmet, Psykiatri Sydväst), regardless of their clinic of origin. A third party, the Karolinska Trial Alliance, have generated the randomization sequence using the digital system TENALEA that is balanced by being built up of blocks of 4 and 6 occurring in random order. The randomization is implemented consecutively in TENALEA, which requires an authorized study coordinator to provide a participant ID to generate a unique allocation certificate with participant ID, allocation, personnel ID and time stamp.

Assessors will be blind to the study objectives as well as group assignment up to the last follow-up. If the blinding is broken, the participant will be assigned another assessor at subsequent follow-ups. The assessors will be asked to guess the intervention received at each assessment after randomization in order control for quality of the blinding. The following study personnel will remain blind to group allocation until the final data analyses are completed: principal investigator, data managers, primary outcome assessors, trial statistician, and health economist.

Interventions

Bergen 4-day treatment (B4DT)

Patients in this arm will receive an intensive treatment delivered in a combined individual and group format (18). The group sizes will be three to six participants with a 1:1 patient to therapist ratio. In the week leading up to the intensive part of the treatment, participants will have two scheduled phone/video calls with a therapist to encourage participants to prepare for the upcoming exposure tasks. Day 1 of the intensive treatment includes psychoeducation about the B4DT-adapted rationale for exposure with response prevention (ERP) and deciding on exposure tasks. Days 2 and 3 focus on individually tailored and therapist-assisted individual ERP

For peer review only - http://bmjopen.bmj.com/site/about/guidelines.xhtml

in as many relevant settings as possible (up to seven hours of ERP per day). In the evenings, patients should continue with self-guided ERP and may receive therapist support via text messages or phone calls on demand. In the afternoon of day 3, patients can invite relatives and friends to a psychoeducation session. Day 4 of the intensive treatment focuses on treatment summary and relapse prevention, as well as planning self-guided ERP for the upcoming three weeks. Fifteen weeks after treatment start, participants have individual follow-up sessions at the clinic where they summarize their experiences after completing treatment. There is no ERP during this session, and it is not a part of the current trial.

All groups will be led by a therapist with expertise in B4DT. All therapists will have participated in the Norwegian OCD training program (13) or have documented equivalent training. Prior to participation, all therapists will have participated in at least two B4DT groups.

Individual CBT

Patients will receive 16 weekly 90-minute sessions (twice weekly during the first two weeks) of individual CBT for OCD with an emphasis on ERP, delivered over 14 weeks according to a validated protocol (12). Sessions 1-2 contain psychoeducation about OCD and CBT, goal setting, and planning of ERP exercises. Sessions 3-14 include therapist-guided ERP (at the clinic, in the patients' homes or elsewhere as needed) with planned self-practice ERP between sessions. Sessions 15-16 contain a summary of the treatment and lessons learned, as well as relapse prevention and planning of continued self-practice ERP.

In this arm, therapists will be licensed psychologists with extensive experience of treating OCD prior to getting involved in the trial. Trainee psychologists under supervision will not be involved in the treatment of participants.

In both arms, inactive patients will be encouraged to engage in the treatment by the clinicians according to the standard clinical routines – such phone calls and letters. Apart from these clinical routines, no extra effort will be made to ensure patients' adherence, to maximize generalizability of the treatment uptake.

Therapist competence and adherence

Therapists in both arms will be licensed clinical psychologists or psychologists under supervision employed at the two specialist OCD clinics. All sessions will be audiotaped, 20% of the recorded sessions will be randomly selected and rated by independent expert psychologists (two for each treatment) not otherwise involved in the project. Adherence to protocol will be rated using checklists developed specifically for the study and for each intervention. Therapist competence will be rated using the Cognitive Therapy Scale–Revised (CTS-R) (20).

For peer review only - http://bmjopen.bmj.com/site/about/guidelines.xhtml

Sample size calculation

A power calculation was conducted to determine the number of participants needed to test the primary hypothesis that B4DT will be non-inferior compared to gold standard CBT at the pre-specified margin of 4 points on the Y-BOCS. There are no specific guidelines on appropriate non-inferiority margins in research on OCD, however a common rule of thumb is half the placebo-controlled effect of gold-standard treatment versus control conditions (21). The chosen margin of 4 is smaller than half of the 10-point effect that has been demonstrated in ERP-trials using Y-BOCS (10). Importantly, from a clinical perspective, a 4-point effect is small enough to consider other factors than symptom reduction, e.g. patient preference, cost effectiveness, and adverse events. Last, this margin gives similar precision compared to previous non-inferiority studies on OCD (22).

Clinical outcome data from a recent randomized controlled trial (23) and regular clinical practice (B4DT) were obtained to simulate study data. The following parameters were used: A pre-treatment mean of 22.6 (SD = 3.78) for both arms, and a post-treatment mean of 12.9 (SD = 4.07) for the gold standard CBT arm. Statistical power was then assessed by simulating 10000 datasets assuming different true group differences. An ANCOVA was then conducted on each dataset to test for between-group differences at post-treatment accounting for pre-treatment scores. With 60 participants per arm, no difference in efficacy between the treatments, and 10% missing values, this test has >95% power to demonstrate non-inferiority (which was considered supported if the upper bound was less than 4). Details on this calculation, including analysis code, is provided in the online pre-registration (https://osf.io/w5bfp/).

Measurements

Table 2 presents the flow of the recruitment and treatment process and lists clinician-rated and self-rated assessments at the different time points.

The primary outcome measure is the blind assessor-rated Y-BOCS, administered at enrollment, at week 4, 7, and 15 (primary endpoint), and at the 7- and 16-month follow-ups (19). The Y-BOCS is a gold-standard instrument for measuring OCD-symptoms having shown excellent interrater reliability (intraclass coefficient of .98) and internal consistency (Chronbach's alpha of .89) (19) as well as good validity (24). Blinded assessors will undergo training prior to conducting clinician-rated assessment and practice on videos of OCD case examples. To be allowed to conduct assessments for this project, assessors will have to deviate no more than 1 point on single items, no more than 2 points on the obsession subscale or the compulsion subscale and no more than 4 points on the Y-BOCS total score compared to expert raters at the two clinics. Inter-rater reliability on the Y-BOCS will be reported.

For peer review only - http://bmjopen.bmj.com/site/about/guidelines.xhtml

Secondary clinician-administered outcome measures are the Clinical Global Impression – Severity and Improvement (CGI-S, CGI-I, blind-rated) (25), the Patient Exposure/Response Prevention Adherence Scale (PEAS) (26), the Structured Clinical Interview for DSM-5 for OCD and related disorders (SCID-5) (27) and the Mini International Neuropsychiatric Interview (28). SCID-5 and MINI will be administered at enrollment to confirm the OCD diagnosis and assess comorbidities.

Secondary self-rated outcome measures are the Obsessive-Compulsive Inventory–Revised (OCI-R) (29), self-rated Y-BOCS (30), Montgomery-Åsberg Depression Rating Scale–Self-Rated (MADRS-S) (31), Work and Social Adjustment Scale (WSAS) (32), Assessing Quality of Life 6 Dimensions (AQoL-6D) (33), Credibility/Expectancy Questionnaire (CEQ) (34), Working Alliance Inventory–Short Form Revised (WAI-SR) (35), the Treatment Inventory of Costs in Psychiatric Patients (TIC-P) resource use questionnaire (for economic evaluation) (36), and the Negative Effects Questionnaire (NEQ) (37). Participants will also rate their treatment preference measured on a 7-point Likert-type scale (pre-treatment and post-treatment). At the end of each treatment, the therapist will provide information on the number of sessions the patient attended as well as any extra time (additional phone calls, supervision, administrative tasks) spent due to the needs of the specific patient.

Table 2. Overview of the recruitment, treatments and assessment points.

	Enrollment	Allocation	Pre-treatment	Week 1-2	Week 4	Week 5-6	Week 7	Week 8-14	Week 15 (primary end-point)	7-month follow-up	16-month follow-up
**Enrollment:**											
Eligibility screen	X										
Informed consent	X										
Allocation		X									
**Treatment**											
Gold-standard CBT				X	X	X	X	X			
B4DT				X							
**Clinician-rated instruments**											
SCID-5 (OCD)	X										
MINI	X										
Y-BOCS	X				X		X		X	X	X
CGI-S	X				X		X		X	X	X
CGI-I					X		X		X	X	X

For peer review only - http://bmjopen.bmj.com/site/about/guidelines.xhtml

PEAS*					X				X		
**Self-rated instruments**											
YBOCS-SR			X						X	X	X
OCI-R			X						X	X	X
MADRS-S			X						X	X	X
WSAS			X						X	X	X
AQoL-6D			X						X	X	X
TIC-P			X						X	X	X
NEQ									X	X	X
Other adverse events					X		X		X	X	X
CEQ**				X							
WAI-SR**				X							
Treatment preference			X						X		

Abbreviations: AQoL-6D, Assessing Quality of Life 6 Dimensions; CEQ, Credibility/Expectancy Questionnaire; CGI-I, Clinical Global Impression–Improvement; CGI-S, Clinical Global Impression–Severity; MADRS-S; Montgomery-Åsberg Depression Rating Scale–Self-Rated; MINI, Mini International Neuropsychiatric Interview; NEQ, Negative Effects Questionnaire; OCI-R, Obsessive-Compulsive Inventory–Revised; PEAS, Patient Exposure/Response Prevention Adherence Scale; SCID-5, Structured Clinical Interview for DSM-5 Disorders; TIC-P, Treatment Inventory of Costs in Psychiatric Patients; WAI-SR, Working Alliance Inventory–Short Form Revised; WSAS, Work and Social Adjustment Scale; Y-BOCS, Yale-Brown Obsessive Compulsive Scale; Y-BOCS-SR, Yale-Brown Obsessive Compulsive Scale–Self-Rated; Other adverse events, semi-structured questions about potential severe adverse events and psychiatric care outside the study

* PEAS rated once at the last day of B4DT treatment in the B4DT-arm

** CEQ and WAI-SR rated by participants at the last day of B4DT treatment and week 2 in individual CBT

Treatment-response will be defined as a ≥35% reduction on the Y-BOCS and a Clinical Global Impression-Improvement (CGI-I) score of 1 (”very much improved”) or 2 (”much improved”). Remission will be defined as a score of ≤12 on the Y-BOCS and a Clinical Global Impression-Severity (CGI-S) rating of 1 (”normal, not at all ill”) or 2 (”borderline mentally ill”). Recovery will be defined as a sustained remission status at the two long-term follow-ups (38). Participants who complete less than 8 sessions of CBT or attend at less than 1.5 days of B4DT will be considered non-completers, unless the treatment is terminated by the therapist due to early recovery.

The participants who do not engage in treatment or drop out from the trial will be encouraged to take part in all outcome assessments, prioritizing the primary outcome measure at the primary end point if the burden of assessments needs to be reduced to increase compliance. In order to assess quality of the data inserted in the electronic case report forms (eCRF), all the

For peer review only - http://bmjopen.bmj.com/site/about/guidelines.xhtml

eCRF:s will be compared to the source data for 10% of the participants be the quality monitor Karolinska Trial Alliance.

Safety and adverse events

Information on adverse events including suicidal ideation, and initiation of additional psychiatric care, will be collected at each assessment following inclusion, as well as through the regular contact between participants and therapists during treatment. The incidents will be handled according to the clinics' routines and described on a case-by-case basis. The NEQ with 32 items on incidents and negative effects will be used at the primary endpoint and follow-ups. Adverse events will be labeled serious if they are life-threatening, result in death or persistent/significant disability/incapacity, require hospitalization or its prolongation, or is considered medically significant by the principal investigator. All the personal data will be stored in locked archive rooms with personal access (analogue data), or on encrypted servers, requiring VPN-connection and/or two-step authentication (digital data) in accordance with Region Stockholm and Karolinska Institutet's data management guidelines.

Statistical analysis

Analysis code and a more detailed analysis plan were uploaded to Open Science Framework before any data had been collected (https://osf.io/w5bfp/, under Preregistration – Analysis).

Primary outcome

The primary non-inferiority hypothesis on the clinician-rated Y-BOCS will be evaluated using an ANCOVA of group differences at week 15 with pre-treatment score as covariate. It will be based on all treatment completers. This per-protocol approach is chosen to avoid potential problems with the intention-to-treat approach in a non-inferiority study design, which can bias the results towards the alternative hypothesis of non-inferiority (39). No data imputation will be conducted at this stage. A 95% confidence interval will be used to model the gold standard CBT vs. B4DT effect. Non-inferiority is supported if the upper bound is less than 4 (the non-inferiority margin). This testing procedure has the Type I error rate controlled at the 2.5% level for both comparisons.

As sensitivity analyses, the same ANCOVA model will be fitted with an intention-to-treat approach, including all randomized participants. Missing data will then be estimated using maximum likelihood after which the models are refitted. The aim of these steps is to address the potential influence of non-random missing data in the primary per protocol analysis.

For peer review only - http://bmjopen.bmj.com/site/about/guidelines.xhtml

Secondary outcomes

The superiority hypothesis (that B4DT is more effective than standard CBT) will be evaluated using the exact same model used for the main non-inferiority hypothesis, see Primary outcome, and superiority will be considered supported if the upper bound of the 95% confidence interval is less than 0.

Speed of response, rates of response and remission, and dropout rates

For other secondary outcomes we will compute 95% confidence intervals of group differences using intent-to-treat approach without comparing the bounds to any predefined margin.Since we focus on pre-to-post-treatment comparisons, no data imputation methods will be therefore used as default (unless stated otherwise). However, if the proportion of missing data exceeds 10%, we will perform maximum likelihood imputations based on demographics and the scores from the pre-treatment assessment. To assess speed of response, responder status in the two treatment arms will be compared at week 4 and 7 using generalized linear model where 95% confidence intervals of the odds ratio of responder status will be reported. The same model will be used to compare responder and remitter status in the groups at week 15 to assess rates of response and remission. Proportions of dropouts in the two treatment groups will be compared using a two-sided two-proportions z-test.

Cost effectiveness

We will conduct a within-trial economic evaluation (40) whereby outcome and cost data will be compared at week 15. It will encompass three analyses: 1) a cost-effectiveness analysis using responder status as outcome; 2) a cost-effectiveness analysis using the remitter status as outcome; and 3) a cost-utility analysis using the outcome quality adjusted life years (QALYs) measured using the AQOL-6D (33).

Health related quality of life (HRQoL) will be collected using the AQOL-6D (33,41), a multiattribute utility instrument for use in economic evaluation. Data on resource use will be collected using an adapted version of the self-reported Treatment Inventory of Costs in Psychiatric Patients (TIC-P) questionnaire (36). Costs will be measured from three perspectives: a healthcare organization payer perspective (including intervention costs only), a healthcare system perspective (additionally including costs related to the use of medical resources, and medication) and a societal perspective (additionally including social care costs and productivity losses for the individual). Costs from each perspective will be analyzed in relation to clinical efficacy (responder status; cost-effectiveness analysis) and QALYs (cost utility analysis). Results will be presented as incremental cost-effectiveness ratios (ICER), as the ratio between the

For peer review only - http://bmjopen.bmj.com/site/about/guidelines.xhtml

difference in costs and the difference in health outcomes ICERs will be put against values of willingness to pay for a QALY to determine cost-effectiveness.

Standard economic evaluation techniques will be used to explore uncertainty around the cost and effect data, which will be represented on cost-effectiveness planes. The probability of cost-effectiveness according to different willingness to pay thresholds will be represented on cost effectiveness acceptability curves (42).

Adverse events

A regression analysis with treatment group as predictor will be used to model the total score from the NEQ at week 15. Additional semi-structured questions about severe adverse events will be reported in a frequency table.

Long-term maintenance of gains

At 7 and 16-month follow-ups we will test both the non-inferiority and superiority hypotheses, as well as assess the rates of response, remission and recovery, cost effectiveness and adverse events. For each outcome measure, we will use the same analysis method as at the primary endpoint described above. The generalized linear model used for rates of response and remission will be applied for rates of recovery.

Additional outcomes used for quality assessment

Therapist competence and adherence

Therapist competence (scored using the Cognitive Therapist Scale–Revised (CTS-R)) and protocol adherence will be presented using descriptive information (e.g., means and standard deviations), as well as inter-rater reliability between independent assessors.

Treatment preference, credibility, working alliance and patient adherence

Responses to the question about treatment preference will be presented descriptively at pre-treatment and week 15 (means and standard deviations). An exploratory analysis will evaluate whether the degree of agreement between pre-treatment preference and treatment received moderates the main Y-BOCS outcome.

Total score means with standard deviations for treatment credibility, working alliance, and patients' adherence will be presented for both treatments.

For peer review only - http://bmjopen.bmj.com/site/about/guidelines.xhtml

Patient and public involvement

Patient representatives have not been involved in the design of the current study. The Swedish OCD Foundation supports the study by providing information about the project to their members and the public through their channels. Results from the study will be reported to the public and relevant patient organizations through open access publishing and lectures.

DISCUSSION

This study is the first trial to directly compare an intensive treatment for OCD, the Bergen 4-day treatment (B4DT) with gold standard individual CBT delivered over 14 weeks. The study is well powered to test whether B4DT is non-inferior to gold standard CBT in terms of symptom reduction with a 4-point non-inferiority margin. If the two treatments are comparable in terms of clinical efficacy, B4DT should be considered a candidate for implementation in regular care as the intensive format may be more suitable or preferable for some patients.

Limitations

First, the two recruitment sites in this study are located in different parts of Stockholm. This means that some participants might prefer to be randomized to the clinic closest to their home, which could pose a risk of selection bias. Second, this study does not have sufficient statistical power to robustly assess the secondary outcomes that will be interpreted as exploratory. Last, therapists and patients will be aware of their allocated treatment arm. However, extensive efforts have been made to improve blinding integrity.

ETHICS AND DISSEMINATION

Patient safety

All patients will receive either gold standard CBT or B4DT delivered by clinicians who are specialized in each of these forms of treatment delivery. Gold standard CBT is already the firsthand choice for this patient group, and its efficacy and safety have been demonstrated in several studies of high quality (10). While B4DT has not yet been evaluated in an RCT with an active control group, it is an intensive face-to-face treatment with an emphasis on ERP. Moreover, uncontrolled studies have suggested that the effect is similar, or even larger, compared to gold standard CBT (15–17). We therefore consider the additional risks associated with participating in the B4DT-arm to be small. All participants will be monitored closely during the study and additional treatment will be provided in case of severe deterioration.

For peer review only - http://bmjopen.bmj.com/site/about/guidelines.xhtml

Participants referred to the clinics by other health-care services who turn out not to be eligible will be offered alternative treatment in regular care. Excluded self-referred participants will be guided to adequate care.

Maximizing knowledge gain and minimizing risk of bias

We will adopt practices that are known to increase the trustworthiness of scientific research such as prospectively registering the study hypotheses and the statistical analysis plan, adhering to gold standard outcome reporting, controlling the risk of false positive results by interpreting the secondary analyses with caution, and sharing materials needed to evaluate our results (the analysis code, the protocol). A detailed description of the dataset will be saved in a searchable database, SND (43). The results of the trial will be communicated in open access scientific publications, at scientific conferences as well as meetings with representatives from health care system and patient organizations, and in public media.

CURRENT TRIAL STATUS

Inclusion started in November 2022 and is expected to end in June 2024. At the time of submission, 47 participants had been randomized. The last follow-up appointment is expected to take place in October 2025. Data analysis and reporting of results will begin when all data from the primary endpoint has been collected (October 2024).

For peer review only

ADDITIONAL INFORMATION

Study sponsor: Region Stockholm, Stockholm Sweden. Contact: managing director Lina Martinsson; lina.martinsson@regionstockholm.se; Psykiatri Sydväst, M 58 Huddinge sjukhusområde, 141 86 Stockholm; 08-123 800 00.

Role of funding sources

This study is partly funded by Helse Bergen, the hospital where B4DT was developed. Additional financial support for the study is provided by CIMED (FoUI-960456), Region Stockholm (ALF project RS2020-0731, ALF Postdoc FoUI-963608) and Stiftelsen Söderström-Königska Sjukhemmet (SLS-981976, SLS-980922).

The funders had no role in study design, data collection and analysis, decision to publish or preparation of the manuscript.

Author contributions

CR initiated the project and CR, VI and EI obtained funding for the trial. EI had the overall responsibility for the project's progression. RF and OF developed the pre-registered analyses plan and drafted this protocol, RF and EI finished it together. FS developed the cost-effectiveness analysis plan. SD, SA, and MS organized the work at the clinics. All authors collaborated on the proposal and methodology and critically reviewed the final version of this protocol.

Personal declaration of interest

Dr Flygare has received speaking fees from the Swedish OCD Association, Insight Events AB, and Kry International AB, as well as reimbursement for writing articles for Inside Practice Psychiatry; all outside the submitted work. Dr Ivanov has received speaking fees from the Swedish OCD Association outside the submitted work. Prof Mataix-Cols receives royalties for contributing articles to UpToDate, Inc., outside the submitted work. Dr Andersson and Ms Asplund receive royalties from Natur & Kultur for books, all outside the submitted work. Dr Rück receives royalties from Natur & Kultur, Studentlitteratur and Albert Bonniers Förlag and has received speaking fees from various sources, all outside the submitted work.

The rest of the authors have nothing to declare.

For peer review only - http://bmjopen.bmj.com/site/about/guidelines.xhtml

REFERENCES

1. American Psychiatric Association. Diagnostic and Statistical Manual of Mental Disorders (DSM-5®). American Psychiatric Pub; 2013. 1520 p.

2. Fontenelle IS, Fontenelle LF, Borges MC, Prazeres AM, Rangé BP, Mendlowicz MV, et al. Quality of life and symptom dimensions of patients with obsessive–compulsive disorder. Psychiatry Research. 2010 Sep;179(2):198–203.

3. Koran LM, Thienemann M, Davenport R. Quality of life for patients with obsessive-compulsive disorder. AJP. 1996 Jun;153(6):783–8.

4. Harvard Medical School. National Comorbidity Survey (NCSSC). Data Table 1: Lifetime prevalence DSM-IV/WMH-CIDI disorders by sex and cohort. [Internet]. 2007 [cited 2017 Aug 21]. Available from: https://www.hcp.med.harvard.edu/ncs/index.php

5. Fernández de la Cruz L, Lichtenstein P, Mataix-Cols D, D'Onofrio BM, Rück C, de la Cruz LF, et al. Suicide in obsessive–compulsive disorder: a population-based study of 36 788 Swedish patients. Mol Psychiatry. 2016 Jul 19;12(April):771–7.

6. Fernández de la Cruz L, Isomura K, Lichtenstein P, Rück C, Mataix-Cols D. Morbidity and mortality in obsessive-compulsive disorder: A narrative review. Neuroscience & Biobehavioral Reviews. 2022 May;136:104602.

7. Pérez-Vigil A, Fernández de la Cruz L, Brander G, Isomura K, Jangmo A, Feldman I, et al. Association of Obsessive-Compulsive Disorder With Objective Indicators of Educational Attainment: A Nationwide Register-Based Sibling Control Study. JAMA Psychiatry. 2018 Jan 1;75(1):47.

8. Pérez-Vigil A, Mittendorfer-Rutz E, Helgesson M, Cruz LF de la, Mataix-Cols D. Labour market marginalisation in obsessive–compulsive disorder: a nationwide register-based sibling control study. Psychological Medicine. 2018 Jun;1–10.

9. Rosa-Alcázar AI, Sánchez-Meca J, Gómez-Conesa A, Marín-Martínez F. Psychological treatment of obsessive-compulsive disorder: a meta-analysis. Clin Psychol Rev. 2008 Dec;28(8):1310–25.

10. Öst LG, Havnen A, Hansen B, Kvale G. Cognitive behavioral treatments of obsessive-compulsive disorder. A systematic review and meta-analysis of studies published 1993-2014. Clin Psychol Rev. 2015 Aug;40:156–69.

11. National Collaborating Centre for Mental Health (UK). Obsessive-Compulsive Disorder: Core Interventions in the Treatment of Obsessive-Compulsive Disorder and Body Dysmorphic Disorder [Internet]. Leicester (UK): British Psychological Society; 2006 [cited 2019 Mar 25]. (National Institute for Health and Clinical Excellence: Guidance). Available from: http://www.ncbi.nlm.nih.gov/books/NBK56458/

12. Foa EB, Yadin E, Lichner TK. Exposure and response (ritual) prevention for obsessive-compulsive disorder: therapist guide. 2nd ed. Oxford ; New York: Oxford University Press; 2012. 182 p. (Treatments that work).

13. Kvale G, Hansen B. Dissemination and Intensifying evidence-based treatment for OCD: Norway is in the lead. The Nordic Psychiatrist. 2014 Jan;3:14–5.

14. Havnen A, Hansen B, Öst LG, Kvale G. Concentrated ERP delivered in a group setting: An effectiveness study. Journal of Obsessive-Compulsive and Related Disorders. 2014 Oct 1;3(4):319–24.

15. Havnen A, Hansen B, Öst LG, Kvale G. Concentrated ERP Delivered in a Group Setting: A Replication Study. Behavioural and Cognitive Psychotherapy. 2017 Sep;45(5):530–6.

For peer review only

For peer review only - http://bmjopen.bmj.com/site/about/guidelines.xhtml

16. Hansen B, Kvale G, Hagen K, Havnen A, Öst LG. The Bergen 4-day treatment for OCD: four years follow-up of concentrated ERP in a clinical mental health setting. Cognitive Behaviour Therapy. 2018 Aug 8;0(0):1–17.

17. Hansen B, Hagen K, Öst LG, Solem S, Kvale G. The Bergen 4-day OCD treatment delivered in a group setting: 12-month follow-up. Front Psychol [Internet]. 2018 [cited 2018 Apr 21];9. Available from: https://www.frontiersin.org/articles/10.3389/fpsyg.2018.00639/abstract

18. Launes G, Hagen K, Sunde T, Öst LG, Klovning I, Laukvik IL, et al. A Randomized Controlled Trial of Concentrated ERP, Self-Help and Waiting List for Obsessive- Compulsive Disorder: The Bergen 4-Day Treatment. Front Psychol [Internet]. 2019 [cited 2019 Nov 19];10. Available from: https://www.frontiersin.org/articles/10.3389/fpsyg.2019.02500/full?utm_source=F-NTF&utm_medium=EMLX&utm_campaign=PRD_FEOPS_20170000_ARTICLE

19. Goodman WK, Price LH, Rasmussen SA, Mazure C, Delgado P, Heninger GR, et al. The Yale-Brown Obsessive Compulsive Scale. I. Development, Use, and Reliability. Arch Gen Psychiatry. 1989 Nov;46(11):1012–6.

20. Blackburn IM, James IA, Milne DL, Baker C, Standart S, Garland A, et al. The Revised Cognitive Therapy Scale (CTS-R): Psychometric Properties. Behav Cogn Psychother. 2001 Oct;29(4):431–46.

21. Committee for medicinal products for human use (CHMP) guideline on the choice of the non-inferiority margin. Statist Med. 2006 May 30;25(10):1628–38.

22. Lovell K, Cox D, Haddock G, Jones C, Raines D, Garvey R, et al. Telephone administered cognitive behaviour therapy for treatment of obsessive compulsive disorder: randomised controlled non-inferiority trial. BMJ. 2006 Oct 28;333(7574):883.

23. Lundström L, Flygare O, Andersson E, Enander J, Bottai M, Ivanov VZ, et al. Effect of Internet-Based vs Face-to-Face Cognitive Behavioral Therapy for Adults With Obsessive-Compulsive Disorder: A Randomized Clinical Trial. JAMA Network Open. 2022 Mar 14;5(3):e221967.

24. Goodman WK. The Yale-Brown Obsessive Compulsive Scale: II. Validity. Arch Gen Psychiatry. 1989 Nov 1;46(11):1012.

25. Guy W (Editor). ECDEU assessment manual for psychopharmacology. Rockville, MD: US Department of Health, Education, and Welfare, Public Health Service, Alcohol, Drug Abuse, and Mental Health Administration; 1976.

26. Simpson HB, Maher M, Page JR, Gibbons CJ, Franklin ME, Foa EB. Development of a Patient Adherence Scale for Exposure and Response Prevention Therapy. Behavior Therapy. 2010 Mar;41(1):30–7.

27. First MB, Williams JBW, Karg RS, Spitzer RL. Structured clinical interview for DSM-5 - Research version. Arlington, VA: American Psychiatric Association; 2015.

28. Sheehan DV, Lecrubier Y, Sheehan KH, Amorim P, Janavs J, Weiller E, et al. The Mini-International Neuropsychiatric Interview (M.I.N.I.): the development and validation of a structured diagnostic psychiatric interview for DSM-IV and ICD-10. J Clin Psychiatry. 1998;59 Suppl 20:22-33;quiz 34-57.

29. Foa EB, Huppert JD, Leiberg S, Langner R, Kichic R, Hajcak G, et al. The Obsessive-Compulsive Inventory: Development and validation of a short version. Psychological Assessment. 2002;14(4):485–96.

30. Steketee G, Frost R, Bogart K. The Yale-Brown Obsessive Compulsive Scale: Interview versus self-report. Behaviour Research and Therapy. 1996 Aug;34(8):675–84.

For peer review only - http://bmjopen.bmj.com/site/about/guidelines.xhtml

31. Svanborg P, Åsberg M. A comparison between the Beck Depression Inventory (BDI) and the self-rating version of the Montgomery Åsberg Depression Rating Scale (MADRS). Journal of Affective Disorders. 2001 May;64(2–3):203–16.

32. Mundt JC, Marks IM, Shear MK, Greist JM. The Work and Social Adjustment Scale: a simple measure of impairment in functioning. Br J Psychiatry. 2002 May;180(5):461–4.

33. Richardson JR, Peacock SJ, Hawthorne G, Iezzi A, Elsworth G, Day NA. Construction of the descriptive system for the assessment of quality of life AQoL-6D utility instrument. Health Qual Life Outcomes. 2012;10(1):38.

34. Devilly GJ, Borkovec TD. Psychometric properties of the credibility/expectancy questionnaire. Journal of Behavior Therapy and Experimental Psychiatry. 2000 Jun;31(2):73–86.

35. Hatcher RL, Gillaspy JA. Development and validation of a revised short version of the working alliance inventory. Psychotherapy Research. 2006 Jan 1;16(1):12–25.

36. Timman R, Bouwmans C, Busschbach JJV, Hakkaart-van Roijen L. Development of the Treatment Inventory of Costs in Psychiatric Patients: TIC-P Mini and Midi. Value in Health. 2015 Dec;18(8):994–9.

37. Rozental A, Kottorp A, Forsström D, Månsson K, Boettcher J, Andersson G, et al. The Negative Effects Questionnaire: psychometric properties of an instrument for assessing negative effects in psychological treatments. 2019;47(5):559–72.

38. Mataix-Cols D, Fernández de la Cruz L, Nordsletten AE, Lenhard F, Isomura K, Simpson HB. Towards an international expert consensus for defining treatment response, remission, recovery and relapse in obsessive-compulsive disorder. World Psychiatry. 2016 Feb;15(1):80–1.

39. Food and Drug Administration. Non-inferiority clinical trials to establish effectiveness: guidance for industry [Internet]. US: Department of Health and Human Services; 2016 [cited 2023 May 11]. Available from: https://www.fda.gov/media/78504/download

40. Drummond M. Methods for the economic evaluation of health care programmes. Fourth edition. Oxford, United Kingdom ; New York, NY, USA: Oxford University Press; 2015. 445 p. (Oxford medical publications).

41. Maxwell A, Özmen M, Iezzi A, Richardson J. Deriving population norms for the AQoL-6D and AQoL-8D multi-attribute utility instruments from web-based data. Qual Life Res. 2016 Dec;25(12):3209–19.

42. Fenwick E, Byford S. A guide to cost-effectiveness acceptability curves. Br J Psychiatry. 2005 Aug;187(2):106–8.

43. Munafò MR, Nosek BA, Bishop DVM, Button KS, Chambers CD, Percie du Sert N, et al. A manifesto for reproducible science. Nat Hum Behav. 2017 Jan 10;1(1):0021.

For peer review only

For peer review only - http://bmjopen.bmj.com/site/about/guidelines.xhtml

FIGURE LEGENDS

Figure 1. CONSORT-flow diagram. Abbreviations: B4DT, Bergen 4-day treatment; CBT, cognitive behavior therapy.

For peer review only

For peer review only - http://bmjopen.bmj.com/site/about/guidelines.xhtml

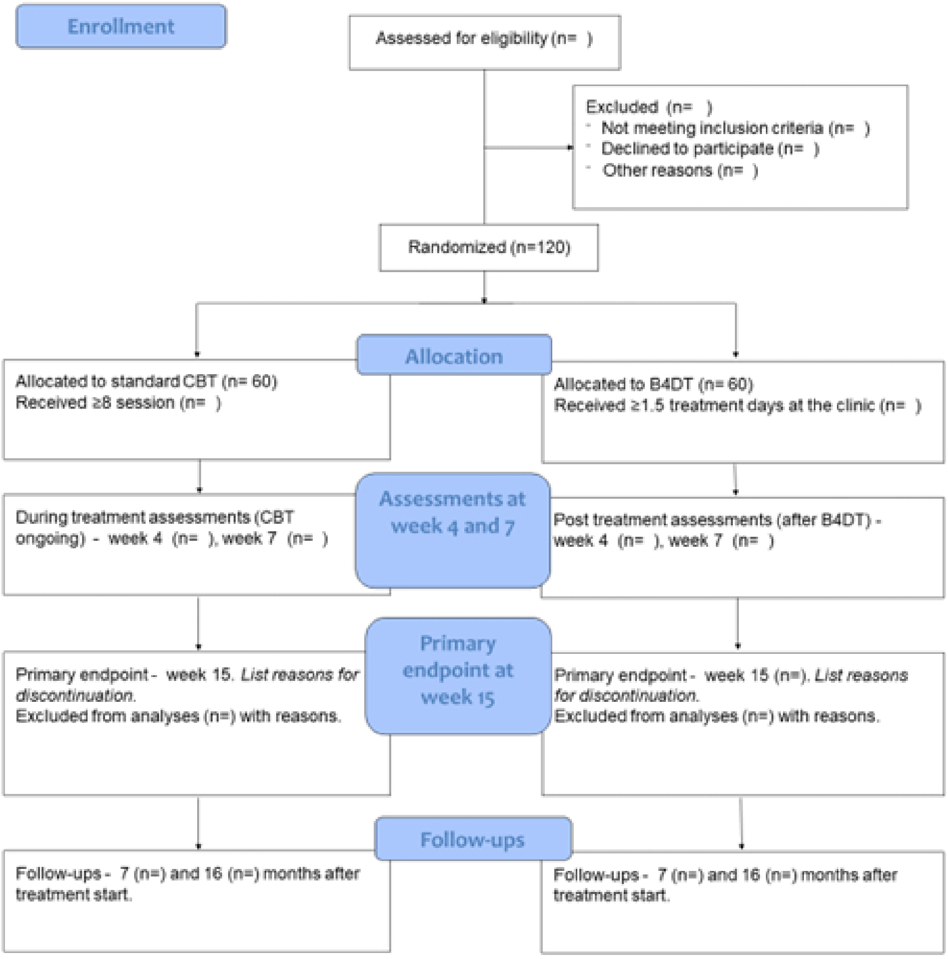

Figure 1. CONSORT-flow diagram. Abbreviations: B4DT, Bergen 4-day treatment; CBT, cognitive behavior therapy.

348x350mm (38 x 38 DPI)

1

Region Stockholm
Stockholms läns sjukvårdsområde

Karolinska Institutet

Intensiv KBT under fyra dagar jämfört med individuell KBT under fyra månader för vuxna med tvångssyndrom: En randomiserad kontrollerad studie

Information till forskningspersoner

Region Stockholm genomför nu en behandlingsstudie för vuxna som lider av tvångssyndrom. I det här dokumentet får du information om projektet och om vad det innebär att delta.

Vad är det för ett projekt och varför vill ni att jag ska delta?

Det finns idag starkt forskningsstöd som visar att kognitiv beteendeterapi (KBT) är en effektiv behandlingsmetod mot tvångssyndrom. Utbudet av KBT i Sverige är begränsat och det är angeläget att hitta nya behandlingsformer så att fler patienter kan erbjudas effektiv vård. KBT ges oftast en gång i veckan under 3-4 månaders tid, ett upplägg som inte passar alla patienter. I den här studien vill vi jämföra KBT med en ny, intensivare variant av KBT som utveckats i Norge och där själva behandlingen ges under endast fyra dagar under en vecka. Om denna nya behandling skulle visa sig vara lika effektiv som KBT skulle den kunna förstärka vårdutbudet för patienter med tvångssyndrom och eventuellt även förkorta sjukdomstiden för dem. Du behöver ha fyllt 18 år för att kunna delta i projektet.

Forskningshuvudmännen för projektet är Region Stockholm och Karolinska Institutet. Med forskningshuvudman menas den organisation som är ansvarig för studien. Studien är godkänd av Etikprövningsmyndigheten, diarienummer 2022-01713-0.

Hur går projektet till?

För att vi ska kunna utvärdera resultaten av behandlingen kommer du före och under behandlingens gång få fylla i flera olika frågeformulär (fyra tillfällen á 30 min). Du får prata med en läkare eller psykolog som kommer ställa frågor som handlar om ditt mående. Uppföljande besök sker även ca 7 månader och 16 månader efter behandlingens start för att vi ska kunna undersöka om effekterna håller i sig.

En förutsättning för att vi ska kunna mäta behandlingens effekt är att alla studiedeltagare slumpas till olika grupper. En grupp får KBT där patienterna träffar sin behandlare en till två gånger i veckan på OCD-programmet på Karolinska sjukhuset i Huddinge, den andra gruppen får intensivbehandlingen på heltid under fyra dagar (exklusive ett par uppföljningssamtal) på Ångestenheten på Karolinska sjukhuset i Solna. Det kommer inte att gå att påverka vilken grupp du kommer att hamna i. Det är därför viktigt att du bara väljer att delta i denna studie om du kan tänka båda behandlingsalternativen och kan resa både till Solna eller Huddinge dagtid för behandling.

Ljudinspelningar från behandlingssessioner och bedömningsbesöken görs för att säkra att terapeuterna och annan personal följer behandlingsprotokollet och bedömningsrutiner. Filerna för ljudinspelning kommer att förstöras efter studiens avslut.

Uppgifter om dig, t.ex. om dina vårdtillfällen, läkemedel och sociala faktorer kommer hämtas ur följande hälsovårds- och befolkningsregister för att studera effekten av behandlingen och dess kostnader: Longitudinell integrationsdatabas för sjukförsäkrings- och arbetsmarknadsstudier (LISA) vid Statistiska Centralbyrån (information om sjukskrivningar och uttag av sjukpenning), Patientregistret vid Socialstyrelsen (avslutade vårdtillfällen i slutenvård, vårdtillfällen hos läkare och inom specialistvården med aktuella diagnoser och sökorsaker, vårdtillfällen inom psykiatrisk tvångsvård), Läkemedelsregistret vid Socialstyrelsen (utskrivna och uthämtade mediciner) och om relevant ett av följande regionala register; Datalager för uppföljning av vårdhändelser i Stockholms Läns Landsting (VAL-databaserna) (vårdtillfällen inom primärvården med aktuella diagnoser och sökorsaker). Registeruppgifterna hämtas för tiden från du startar i studien till 16 månader efter påbörjad behandling.

For peer review only

Region Stockholm
Stockholms läns sjukvårdsområde

Karolinska Institutet

Behandlingen är kostnadsfri. Som deltagare i studien kommer du antingen delta i en fjorton veckor lång behandling, eller i en intensiv fyradagarsbehandling. För att vara aktuell för studien krävs därför att du har:

- *möjlighet att ta dig till Huddinge Sjukhus 1-2 gånger/vecka under behandlingstiden på 14 veckor.*
- *möjlighet att ta dig till och vara på Karolinska Sjukhuset i Solna under 4 heldagar under en vecka, samt delta vid två introduktionstillfällen veckan.*

Möjliga följder och risker med att delta i projektet

Genom att delta ger du oss möjlighet att studera hur det går i behandlingen vilket kan leda till förbättringar i framtida vård. Du kommer inte att få betalt för att delta. Målet med bägge behandlingar är att du ska ha mindre symtom av tvångssyndrom efter avslutad behandling. Vägen dit kan innebära starka känslor och behandlarna kommer hjälpa dig hantera dessa. Du kommer behöva lägga mycket tid på behandlingen.

Vad händer med mina uppgifter?

Projektet kommer att samla in information om dig. Uppgifter som kan kopplas till dig som person kommer att lagras och hanteras enligt särskilda rutiner som godkänts av huvudmännen. En kodlista som förvaras separat kommer att upprättas där dina personuppgifter tilldelas ett studie-ID. Dessa uppgifter lagras enligt gällande riktlinjer för forskningsdokumentation. Dina svar och dina resultat kommer att behandlas så att obehöriga inte kan ta del av dem. Dina sjukvårdskontakter journalförs på vanligt sätt enligt sjukvårdens rutiner. SLSO kommer att lämna ut kodade (pseudonymiserade) svar på de frågeformulär du besvarar till forskarna på Karolinska Institutet som analyserar resultaten.

SLSO och Karolinska Institutet är båda forskningshuvudmän för denna studie och därmed ansvariga för behandlingen av dina personuppgifter. Din kontaktperson för denna behandling är huvudansvarig forskare Christian Rück. Dina personuppgifter kommer i första hand att behandlas av huvudansvarig forskare och hans medarbetare, men kan även efter särskild prövning komma lämnas ut till andra forskare i syfte att besvara andra frågeställningar. All hantering av din data kommer att ske i forskningssyfte och regleras av EUs dataskyddsförordning (GDPR) utifrån den rättsliga grunden att utföra en uppgift av allmänt intresse.

Vi använder uppgifterna vi samlar in om dig för att kunna utvärdera behandlingen. Dina uppgifter sparas i minst 10 år. Du har rätt att en gång årligen begära ut ett registerutdrag på de uppgifter som lagras om dig. Enligt EU:s dataskyddsförordning har du rätt att kostnadsfritt få ta del av de uppgifter om dig som hanteras i projektet, och vid behov få eventuella fel rättade. Du kan också begära att uppgifter om dig raderas samt att behandlingen av dina personuppgifter begränsas. Rätten till radering och till begränsning av behandling av personuppgifter gäller dock inte när uppgifterna är nödvändiga för den aktuella forskningen. Vid denna typ av ärenden ska du kontakta huvudansvarig forskare.

Om du har synpunkter på behandlingen av dina personuppgifter kan du vända dig till SLSOs dataskyddsombud på gdpr.slso@sll.se. Om du inte är nöjd med SLSOs svar, kan du vända dig till Integritetsskyddsmyndigheten med klagomål imy@imy.se eller 08-657 61 00.

3

Region Stockholm
Stockholms läns sjukvårdsområde

Karolinska Institutet

Hur får jag information om resultatet av projektet?

Efter studiens avslut kommer du att kunna ta del av resultatet i form av en vetenskaplig publikation. Varken du eller någon annan kommer där kunna identifiera vilka datapunkter som är dina.

Försäkring och ersättning

Alla patienter och friska frivilliga som deltar i en klinisk studie, utförd inom svensk hälso- och sjukvård, täcks av en patientförsäkring via patientskadelagen. (SFS nr 1996:799). Ingen ersättning för deltagande i studien ges.

Deltagandet är frivilligt

Ditt deltagande i studien är frivilligt och du kan när som helst välja att avbryta deltagandet. Om du vill avbryta ditt deltagande behöver du inte uppge varför, och det kommer inte heller att påverka din framtida vård eller behandling. I det fall du väljer att inte delta, eller av någon anledning skulle välja att avbryta ett påbörjat deltagande, kommer våra behandlare att hänvisa dig till annan lämplig psykiatrisk vård. Om du vill avbryta ditt deltagande ska du kontakta den ansvariga för projektet (se nedan).

Ansvariga för projektet

Christian Rück, professor, överläkare
Huvudansvarig forskare
Telefonnummer: 0704843392
e-post: christian.ruck@ki.se

Ekaterina Ivanova, psykolog
Projektledare
Telefonnummer: 0765807416
e-post: ekaterina.ivanova@ki.se

Samtycke till deltagande

Jag har fått muntlig och/eller skriftlig information om studien och har haft möjlighet att ställa frågor. Jag får behålla den skriftliga informationen.

Jag samtycker till att delta i projektet **Intensiv KBT under fyra dagar jämfört med individuell KBT under fyra månader för vuxna med tvångssyndrom: En randomiserad kontrollerad studie**

Ort…………………… Datum ……………………

Namn (textat) ……………………………………………………… Namnteckning ………………………………………………………

Personnummer ………………………………………………………

Upprättas i två exemplar varav originalet arkiveras av ansvarig prövare och forskningspersonen får en kopia.

For peer review only

World Health Organization Trial Registration Data Set

Study protocol for a single-blind, parallel-group, randomized, controlled non-inferiority trial of four-day intensive versus standard cognitive behavioral therapy for adults with obsessive-compulsive disorder.

Data category	Information[32]
Primary registry and trial identifying number	ClinicalTrials.gov<br>NCT05608278
Date of registration in primary registry	8 November, 2022
Secondary identifying numbers	Open Science Framework: https://osf.io/w5bfp/
Source(s) of monetary or material support	This study is partly funded by Helse Bergen, the hospital where B4DT was developed. Additional financial support for the study is provided by CIMED (FoUI-960456), Region Stockholm (ALF project RS2020-0731, ALF Postdoc FoUI-963608) and Stiftelsen Söderström-Königska Sjukhemmet (SLS-981976, SLS-980922).
Primary sponsor	Region Stockholm, Sweden
Secondary sponsor(s)	Karolinska Institutet, Sweden
Contact for public queries	Dr Ekaterina Ivanova, Region Stockholm and Karolinska Institutet, 0046765807416, ekaterina.ivanova@ki.se
Contact for scientific queries	Dr Ekaterina Ivanova, Region Stockholm and Karolinska Institutet, 0046765807416, ekaterina.ivanova@ki.se
Public title	Study protocol for a single-blind, parallel-group, randomized, controlled non-inferiority trial of four-day intensive versus standard cognitive behavioral therapy for adults with obsessive-compulsive disorder
Scientific title	Study protocol for a single-blind, parallel-group, randomized, controlled non-inferiority trial of four-day intensive versus standard cognitive behavioral therapy for adults with obsessive-compulsive disorder
Countries of recruitment	Sweden
Health condition(s) or problem(s) studied	Obsessive-Compulsive Disorder
Intervention(s)	Active comparator: *The Bergen 4-day treatment*<br>Control comparator: Gold-standard CBT during 14 weeks
Key inclusion and exclusion criteria	Ages eligible for study: ≥18 years<br>Sexes eligible for study: both<br>Accepts healthy volunteers: no<br><br>Inclusion criteria:<br>≥ 18 years of age.

For peer review only

	• Primary diagnosis of OCD according to DSM-5.<br>• Clinician-rated Y-BOCS score of ≥ 16.<br>• Written informed consent.<br>• Being willing and able to attend treatment at any one of the two treatment clinics, regardless of the clinic where the initial assessment took place (the two clinics are located at different locations in Stockholm, about 20 km apart).<br>• Being fluent in Swedish.<br><br>Exclusion criteria:<br><br>• Other psychological treatment for OCD planned during the trial period.<br>• Completed CBT with exposure and response prevention (ERP) for OCD in the last 12 months.<br>• Changes in psychotropic medication within the last 2 months.<br>• Bipolar disorder.<br>• Psychosis.<br>• Alcohol or substance dependence.<br>• Organic brain disorder.<br>• Hoarding disorder or OCD with primary hoarding symptoms.<br>• Suicidal ideation that would warrant close monitoring.
Study type	Interventional<br>Allocation: randomized<br>Intervention model: parallel assignment<br>Masking: single blind (outcomes assessor)<br>Primary purpose: treatment<br>Phase NA
Date of first enrolment	November 2022
Target sample size	120
Recruitment status	Recruiting
Primary outcome(s)	We will compare the two groups 15 weeks after treatment start (primary endpoint) with regard to OCD severity. We hypothesize that B4DT will be non-inferior to standard CBT using a non-inferiority margin of 4 points on the Yale-Brown Obsessive-Compulsive Scale (Y-BOCS) (primary outcome).
Key secondary outcomes	1) Speed of response<br>2) Rates of response and remission<br>3) Dropout rate<br>4) Cost-effectiveness<br>5) Adverse events

For peer review only

For peer review only

STANDARD PROTOCOL ITEMS: RECOMMENDATIONS FOR INTERVENTIONAL TRIALS

SPIRIT 2013 Checklist: Recommended items to address in a clinical trial protocol and related documents*

Section/item	Item No	Description	Addressed on page number
**Administrative information**			
Title	1	Descriptive title identifying the study design, population, interventions, and, if applicable, trial acronym	1
Trial registration	2a	Trial identifier and registry name. If not yet registered, name of intended registry	2
	2b	All items from the World Health Organization Trial Registration Data Set	Appendix 2
Protocol version	3	Date and version identifier	1
Funding	4	Sources and types of financial, material, and other support	16
Roles and responsibilities	5a	Names, affiliations, and roles of protocol contributors	1
	5b	Name and contact information for the trial sponsor	16
	5c	Role of study sponsor and funders, if any, in study design; collection, management, analysis, and interpretation of data; writing of the report; and the decision to submit the report for publication, including whether they will have ultimate authority over any of these activities	16
	5d	Composition, roles, and responsibilities of the coordinating centre, steering committee, endpoint adjudication committee, data management team, and other individuals or groups overseeing the trial, if applicable (see Item 21a for data monitoring committee)	4, 6, 10

For peer review only

**Introduction**			
Background and rationale	6a	Description of research question and justification for undertaking the trial, including summary of relevant studies (published and unpublished) examining benefits and harms for each intervention	3
	6b	Explanation for choice of comparators	3
Objectives	7	Specific objectives or hypotheses	4
Trial design	8	Description of trial design including type of trial (eg, parallel group, crossover, factorial, single group), allocation ratio, and framework (eg, superiority, equivalence, noninferiority, exploratory)	4
**Methods: Participants, interventions, and outcomes**			
Study setting	9	Description of study settings (eg, community clinic, academic hospital) and list of countries where data will be collected. Reference to where list of study sites can be obtained	4
Eligibility criteria	10	Inclusion and exclusion criteria for participants. If applicable, eligibility criteria for study centres and individuals who will perform the interventions (eg, surgeons, psychotherapists)	5-6, 7
Interventions	11a	Interventions for each group with sufficient detail to allow replication, including how and when they will be administered	6-7, 9-10
	11b	Criteria for discontinuing or modifying allocated interventions for a given trial participant (eg, drug dose change in response to harms, participant request, or improving/worsening disease)	11
	11c	Strategies to improve adherence to intervention protocols, and any procedures for monitoring adherence (eg, drug tablet return, laboratory tests)	7, 10
	11d	Relevant concomitant care and interventions that are permitted or prohibited during the trial	5
Outcomes	12	Primary, secondary, and other outcomes, including the specific measurement variable (eg, systolic blood pressure), analysis metric (eg, change from baseline, final value, time to event), method of aggregation (eg, median, proportion), and time point for each outcome. Explanation of the clinical relevance of chosen efficacy and harm outcomes is strongly recommended	8-11
Participant timeline	13	Time schedule of enrolment, interventions (including any run-ins and washouts), assessments, and visits for participants. A schematic diagram is highly recommended (see Figure)	9-10

For peer review only - http://bmjopen.bmj.com/site/about/guidelines.xhtml

Sample size	14	Estimated number of participants needed to achieve study objectives and how it was determined, including clinical and statistical assumptions supporting any sample size calculations	8
Recruitment	15	Strategies for achieving adequate participant enrolment to reach target sample size	5

Methods: Assignment of interventions (for controlled trials)

Allocation:

Sequence generation	16a	Method of generating the allocation sequence (eg, computer-generated random numbers), and list of any factors for stratification. To reduce predictability of a random sequence, details of any planned restriction (eg, blocking) should be provided in a separate document that is unavailable to those who enrol participants or assign interventions	6
Allocation concealment mechanism	16b	Mechanism of implementing the allocation sequence (eg, central telephone; sequentially numbered, opaque, sealed envelopes), describing any steps to conceal the sequence until interventions are assigned	6
Implementation	16c	Who will generate the allocation sequence, who will enrol participants, and who will assign participants to interventions	6
Blinding (masking)	17a	Who will be blinded after assignment to interventions (eg, trial participants, care providers, outcome assessors, data analysts), and how	6
	17b	If blinded, circumstances under which unblinding is permissible, and procedure for revealing a participant's allocated intervention during the trial	NA

Methods: Data collection, management, and analysis

Data collection methods	18a	Plans for assessment and collection of outcome, baseline, and other trial data, including any related processes to promote data quality (eg, duplicate measurements, training of assessors) and a description of study instruments (eg, questionnaires, laboratory tests) along with their reliability and validity, if known. Reference to where data collection forms can be found, if not in the protocol	10-11
	18b	Plans to promote participant retention and complete follow-up, including list of any outcome data to be collected for participants who discontinue or deviate from intervention protocols	10

Data management	19	Plans for data entry, coding, security, and storage, including any related processes to promote data quality (eg, double data entry; range checks for data values). Reference to where details of data management procedures can be found, if not in the protocol	10
Statistical methods	20a	Statistical methods for analysing primary and secondary outcomes. Reference to where other details of the statistical analysis plan can be found, if not in the protocol	11-14
	20b	Methods for any additional analyses (eg, subgroup and adjusted analyses)	11-14
	20c	Definition of analysis population relating to protocol non-adherence (eg, as randomised analysis), and any statistical methods to handle missing data (eg, multiple imputation)	11-14
**Methods: Monitoring**			
Data monitoring	21a	Composition of data monitoring committee (DMC); summary of its role and reporting structure; statement of whether it is independent from the sponsor and competing interests; and reference to where further details about its charter can be found, if not in the protocol. Alternatively, an explanation of why a DMC is not needed	4, 6, 10
	21b	Description of any interim analyses and stopping guidelines, including who will have access to these interim results and make the final decision to terminate the trial	NA
Harms	22	Plans for collecting, assessing, reporting, and managing solicited and spontaneously reported adverse events and other unintended effects of trial interventions or trial conduct	11
Auditing	23	Frequency and procedures for auditing trial conduct, if any, and whether the process will be independent from investigators and the sponsor	4, 6, 10
**Ethics and dissemination**			
Research ethics approval	24	Plans for seeking research ethics committee/institutional review board (REC/IRB) approval	4-5
Protocol amendments	25	Plans for communicating important protocol modifications (eg, changes to eligibility criteria, outcomes, analyses) to relevant parties (eg, investigators, REC/IRBs, trial participants, trial registries, journals, regulators)	5

For peer review only - http://bmjopen.bmj.com/site/about/guidelines.xhtml

Consent or assent	26a	Who will obtain informed consent or assent from potential trial participants or authorised surrogates, and how (see Item 32)	5
	26b	Additional consent provisions for collection and use of participant data and biological specimens in ancillary studies, if applicable	NA
Confidentiality	27	How personal information about potential and enrolled participants will be collected, shared, and maintained in order to protect confidentiality before, during, and after the trial	11
Declaration of interests	28	Financial and other competing interests for principal investigators for the overall trial and each study site	NA
Access to data	29	Statement of who will have access to the final trial dataset, and disclosure of contractual agreements that limit such access for investigators	15
Ancillary and post-trial care	30	Provisions, if any, for ancillary and post-trial care, and for compensation to those who suffer harm from trial participation	11
Dissemination policy	31a	Plans for investigators and sponsor to communicate trial results to participants, healthcare professionals, the public, and other relevant groups (eg, via publication, reporting in results databases, or other data sharing arrangements), including any publication restrictions	15
	31b	Authorship eligibility guidelines and any intended use of professional writers	15
	31c	Plans, if any, for granting public access to the full protocol, participant-level dataset, and statistical code	2, 11
**Appendices**			
Informed consent materials	32	Model consent form and other related documentation given to participants and authorised surrogates	Appendix 1
Biological specimens	33	Plans for collection, laboratory evaluation, and storage of biological specimens for genetic or molecular analysis in the current trial and for future use in ancillary studies, if applicable	NA

*It is strongly recommended that this checklist be read in conjunction with the SPIRIT 2013 Explanation & Elaboration for important clarification on the items. Amendments to the protocol should be tracked and dated. The SPIRIT checklist is copyrighted by the SPIRIT Group under the Creative Commons "Attribution-NonCommercial-NoDerivs 3.0 Unported" license.

For peer review only - http://bmjopen.bmj.com/site/about/guidelines.xhtml

BMJ Open

Study protocol for a single-blind, parallel-group, randomized, controlled non-inferiority trial of four-day intensive versus standard cognitive behavioral therapy for adults with obsessive-compulsive disorder

Journal:	*BMJ Open*
Manuscript ID	bmjopen-2023-076361.R1
Article Type:	Protocol
Date Submitted by the Author:	13-Oct-2023
Complete List of Authors:	Ivanova, Ekaterina; Karolinska Institutet, Center for Psychiatry Research, Department of Clinical Neuroscience; Region Stockholm, Stockholm Health Care Services<br>Fondberg, Robin; Karolinska Institutet, Centre for Psychiatry Research, Department of Clinical Neuroscience<br>Flygare, Oskar; Karolinska Institutet, Center for Psychiatry Research, Department for Clinical Neuroscience; Region Stockholm, Stockholm Health Care Services<br>Sannemalm, Max; Karolinska Institutet, Centre for Psychiatry Research, Department of Clinical Neuroscience; Region Stockholm, Stockholm Health Care Services<br>Asplund, Sofia; Region Stockholm, Stockholm Health Care Services<br>Dahlén, Sofia; Region Stockholm, Stockholm Health Care Services<br>Sampaio, Filipa; Uppsala Universitet, Department of Public Health and Caring Sciences<br>Andersson, Erik; Karolinska Institutet, Division of Psychology, Department of Clinical Neuroscience<br>Mataix-Cols, David; Karolinska Institutet, Centre for Psychiatry Research, Department of Clinical Neuroscience; Region Stockholm, Stockholm Health Care Services<br>Ivanov, Volen; Karolinska Institutet, Centre for Psychiatry Research, Department of Clinical Neuroscience; Region Stockholm, Stockholm Health Care Services<br>Rück, Christian; Karolinska Institutet, Centre for Psychiatry Research, Department of Clinical Neuroscience; Region Stockholm, Stockholm Health Care Services
**Primary Subject Heading**:	Mental health
Secondary Subject Heading:	Mental health
Keywords:	Adult psychiatry < PSYCHIATRY, Anxiety disorders < PSYCHIATRY, PSYCHIATRY

Study protocol for a single-blind, parallel-group, randomized, controlled non-inferiority trial of four-day intensive versus standard cognitive behavioral therapy for adults with obsessive-compulsive disorder

Document date: 13/10/2023
Document version: 1.2
Word count: 3,978

Authors: Ekaterina Ivanova[1]*, Robin Fondberg[2]*, Oskar Flygare[1], Max Sannemalm[1], Sofia Asplund[3], Sofia Dahlén[3], Filipa Sampaio[4], Erik Andersson[5], David Mataix-Cols[1,6], Volen Ivanov[1], Christian Rück[1]

**=contributed equally*

Author affiliations:

[1] Centre for Psychiatry Research, Department of Clinical Neuroscience, Karolinska Institutet, & Stockholm Health Care Services, Region Stockholm, Sweden

[2] Centre for Psychiatry Research, Department of Clinical Neuroscience, Karolinska Institutet, Sweden

[3] Stockholm Health Care Services, Region Stockholm, Sweden

[4] Department of Public Health and Caring Sciences, Uppsala University, Uppsala, Sweden

[5] Division of Psychology, Department of Clinical Neuroscience, Karolinska Institutet, Sweden

[6] Department of Clinical Sciences, Lund University, Lund, Sweden

Corresponding author: Ekaterina Ivanova, ekaterina.ivanova@ki.se, Huddinge sjukhusområde M46, Medicingatan 6, plan 4, 141 86 STOCKHOLM

For peer review only
For peer review only - http://bmjopen.bmj.com/site/about/guidelines.xhtml

ARTICLE SUMMARY

Abstract

Introduction

Individual cognitive behavioral therapy (CBT) with exposure and response prevention (ERP) is an effective treatment for obsessive-compulsive disorder (OCD). However, individual CBT is costly and time-consuming, requiring weekly therapy sessions for 3 to 4 months. A 4-day intensive version of CBT for OCD delivered in group format has been recently developed in Norway (Bergen 4-Day Treatment, B4DT). B4DT has shown promising results in several uncontrolled and one small, randomized trial, but its non-inferiority to the gold-standard treatment has not been established.

Methods and analysis

This single-blind, randomized controlled trial including 120 patients (60 per arm) will compare B4DT to individual CBT. The primary outcome is the blind assessor-rated Yale-Brown Obsessive-Compulsive Scale (Y-BOCS). We hypothesize that B4DT will be non-inferior to gold standard CBT 15 weeks after treatment start. The non-inferiority margin is set at 4 points on the Y-BOCS. Secondary outcomes include time to treatment response, cost effectiveness, response and remission rates, dropout rates, and adverse events.

Ethics and dissemination

This study has been approved by the Swedish Ethical Review Authority. Hypotheses were specified and analysis code published before data collection started. Results from all analyses will be reported in accordance with the Consolidated Standards of Reporting Trials statement for non-pharmacological trials (CONSORT) and Consolidated Health Economic Evaluation Reporting Standards (CHEERS) irrespective of outcome.

Trial registration number

Clinicaltrials.gov; NCT05608278. Open Science Framework; https://osf.io/w5bfp/.

Keywords: Obsessive-compulsive disorder, cognitive behavior therapy, intensive treatment

For peer review only - http://bmjopen.bmj.com/site/about/guidelines.xhtml

Strengths and limitations

- High statistical power to test the primary hypothesis that B4DT is not more than 4 points worse on Y-BOCS than gold standard CBT 15 weeks after treatment start.
- Prespecified methodology and analysis code.
- The blind assessors are blinded to the entire study design.
- Low statistical power to robustly evaluate the secondary outcomes.
- Participants will receive treatment at clinics located in different parts of Stockholm, which may be an obstacle for recruitment and retention.

INTRODUCTION

Obsessive-compulsive disorder (OCD), is characterized by recurrent uncontrollable (obsessions), and repetitive behaviors (compulsions) (1), is highly disabling (2,3), has a lifetime prevalence of 2-3% (4) and is associated with increased morbidity and mortality (5,6), academic underachievement (7) and labor-market marginalization (8).

Cognitive behavioral therapy (CBT) with exposure and response prevention (ERP) is a well-established treatment for OCD (9,10), recommended by international guidelines as a first-line treatment for the disorder (11). CBT is most commonly delivered in an individual format with weekly sessions during three to four months (12). This means that patients experience a relatively slow road to recovery. If treatment could be delivered in a more intensive way, for a shorter period, without sacrificing clinical efficacy, patients could return to their normal lives earlier. Furthermore, this may potentially result in more cost-effective use of clinical resources.

Researchers in Norway have recently developed an intensive version of CBT for OCD - the Bergen 4-Day Treatment (B4DT) (13). Results from uncontrolled trials indicate that approximately 90% of patients reliably improved (14–17) and that 70% were in remission four years after treatment (16). A recent RCT (n=48) showed that B4DT was superior both to a self-help book for OCD and a wait list control (18). Thus, B4DT is potentially effective and attractive for patients and caregivers alike, but its efficacy and cost-effectiveness have not yet been investigated in a head-to-head comparison with gold-standard individual CBT.

In this single-blind, non-inferiority randomized controlled trial, we will evaluate the efficacy of B4DT compared to gold standard, individual CBT. If, as we hypothesize, B4DT is non-inferior to traditional CBT, it could expand the range of available evidence-based options for this patient group.

For peer review only - http://bmjopen.bmj.com/site/about/guidelines.xhtml

Aim and objectives

The primary objective of the study is to evaluate if the intensive Bergen 4-day treatment (B4DT) is a non-inferior alternative to gold standard individual cognitive behavioral therapy (CBT) for reducing symptom severity in adults with OCD.

We will compare the two groups 15 weeks after treatment start (primary endpoint) with regard to OCD severity. We hypothesize that B4DT will be non-inferior to standard CBT using a non-inferiority margin of 4 points on the Yale-Brown Obsessive-Compulsive Scale (Y-BOCS) (19).

Our secondary objectives are:

1) (Primary endpoint) To test whether B4DT is superior to standard CBT
2) (Primary endpoint) To compare B4DT and gold standard CBT with regards to:
    a. Speed of response
    b. Rates of response and remission
    c. Dropout rate
    d. Cost-effectiveness
    e. Adverse events

    Due to its short and intensive format, we expect B4DT to provide a faster response than gold-standard CBT. For secondary objective 2.b-2.e, we have no directed hypotheses. As this study is not sufficiently powered to make confirmatory claims based on the results of these secondary analyses, results will be interpreted as exploratory.

3) Long-term maintenance of gains

METHODS

Study Design

We will conduct a single-blind (blinded outcome assessors), randomized (1:1), controlled, parallel-group, non-inferiority trial comparing intensive 4-day CBT (B4DT) and gold standard individual CBT for adults with OCD residing in Sweden. A total of 120 participants will be assessed before treatment start, at week 4, 7, and 15 (primary endpoint), and 7- and 16 months after treatment start (follow-ups). The Consolidated Standards of Reporting Trials (CONSORT) flow chart of the trial is shown in Figure 1. The outcome measures, statistical models and cut-offs, time-points of evaluation, and analysis code were published on osf.io (https://osf.io/w5bfp/) before data collection began. The study will follow Good Clinical Practice (GCP) and all quality- and safety aspects will be regularly monitored by an external party, the Karolinska Trial Alliance. The study has been approved by the Swedish Ethical Review

For peer review only - http://bmjopen.bmj.com/site/about/guidelines.xhtml

Authority (EPM 2022-01713-01). Potential protocol modifications will be described in detail on osf.io, clinicaltrials.org, and – if the modification touches upon an ethical issue – an amendment will be submitted to the Swedish Ethical Review Authority.

[Figure 1 at about here]

Participants

Patients referred to one of the two involved OCD specialist clinics in Stockholm and self-referred patients will be assessed for eligibility by a staff clinician during a full psychiatric assessment. Candidates that live within approximately one hour of both clinics and agree to participate irrespective of allocation site will be offered inclusion if they match the inclusion/exclusion criteria (see Table 1). Informed consent will either be obtained during a face-to-face clinician visit or on an encrypted study platform, requiring identification and two-step authentication. Once the patient is included in the trial, data on any concurrent treatments will be collected, but those are not prohibited and will not automatically lead to discontinuation of the patient's participation. Karolinska trial alliance is planning a total of seven visits to the study sites, during which they will monitor presence of signed informed consent, documentation of inclusion/exclusion criteria and randomization for each participant.

**Table 1. Overview of inclusion and exclusion criteria**	
**Inclusion criteria**	≥ 18 years of age.
	Primary diagnosis of OCD according to DSM-5.
	Clinician-rated Y-BOCS score of ≥ 16.
	Written informed consent.
	Being willing and able to attend treatment at any one of the two treatment clinics, regardless of the clinic where the initial assessment took place (the two clinics are located at different locations in Stockholm, about 20 km apart).
	Being fluent in Swedish.
**Exclusion criteria**	Other psychological treatment for OCD planned during the trial period.
	Completed CBT with exposure and response prevention (ERP) for OCD in the last 12 months.
	Changes in psychotropic medication within the last 2 months.
	Bipolar disorder.
	Psychosis.

For peer review only - http://bmjopen.bmj.com/site/about/guidelines.xhtml

	Alcohol or substance dependence.
	Organic brain disorder.
	Hoarding disorder or OCD with primary hoarding symptoms.
	Suicidal ideation that would warrant close monitoring.
Abbreviations: CBT, cognitive behavior therapy; DSM-5, The Diagnostic and Statistical Manual of Mental Disorders, Fifth Edition; OCD, obsessive-compulsive disorder; Y-BOCS, Yale-Brown Obsessive-Compulsive Scale.	

For peer review only

Randomization and concealment

The included participants are randomized consecutively to receive either B4DT (delivered at Ångestenheten, Psykiatri Nordväst), or gold standard CBT (delivered at OCD-programmet, Psykiatri Sydväst), regardless of their clinic of origin. A third party, the Karolinska Trial Alliance, have generated the randomization sequence using the digital system TENALEA that is balanced by being built up of blocks of 4 and 6 occurring in random order. The randomization is implemented consecutively in TENALEA, which requires an authorized study coordinator to provide a participant ID to generate a unique allocation certificate with participant ID, allocation, personnel ID and time stamp.

Assessors will be blind to the study objectives as well as group assignment up to the last follow-up. If the blinding is broken, the participant will be assigned another assessor at subsequent follow-ups. The assessors will be asked to guess the intervention received at each assessment after randomization in order control for quality of the blinding. The following study personnel will remain blind to group allocation until the final data analyses are completed: principal investigator, data managers, primary outcome assessors, trial statistician, and health economist.

Interventions

Bergen 4-day treatment (B4DT)

Patients in this arm will receive an intensive treatment delivered in a combined individual and group format (18). The group sizes will be three to six participants with a 1:1 patient to therapist ratio. In the week leading up to the intensive part of the treatment, participants will have two scheduled phone/video calls with a therapist to encourage participants to prepare for the upcoming exposure tasks. Day 1 of the intensive treatment includes psychoeducation about the B4DT-adapted rationale for exposure with response prevention (ERP) and deciding on exposure tasks. Days 2 and 3 focus on individually tailored and therapist-assisted individual ERP

For peer review only - http://bmjopen.bmj.com/site/about/guidelines.xhtml

in as many relevant settings as possible (up to seven hours of ERP per day). In the evenings, patients should continue with self-guided ERP and may receive therapist support via text messages or phone calls on demand. In the afternoon of day 3, patients can invite relatives and friends to a psychoeducation session. Day 4 of the intensive treatment focuses on treatment summary and relapse prevention, as well as planning self-guided ERP for the upcoming three weeks. Fifteen weeks after treatment start, participants have individual follow-up sessions at the clinic where they summarize their experiences after completing treatment. There is no ERP during this session, and it is not a part of the current trial.

All groups will be led by a therapist with expertise in B4DT. All therapists will have participated in the Norwegian OCD training program (13) or have documented equivalent training. Prior to participation, all therapists will have participated in at least two B4DT groups.

Individual CBT

Patients will receive 16 weekly 90-minute sessions (twice weekly during the first two weeks) of individual CBT for OCD with an emphasis on ERP, delivered over 14 weeks according to a validated protocol (12). Sessions 1-2 contain psychoeducation about OCD and CBT, goal setting, and planning of ERP exercises. Sessions 3-14 include therapist-guided ERP (at the clinic, in the patients' homes or elsewhere as needed) with planned self-practice ERP between sessions. Sessions 15-16 contain a summary of the treatment and lessons learned, as well as relapse prevention and planning of continued self-practice ERP.

In this arm, therapists will be licensed psychologists with extensive experience of treating OCD prior to getting involved in the trial. Trainee psychologists under supervision will not be involved in the treatment of participants.

In both arms, inactive patients will be encouraged to engage in the treatment by the clinicians according to the standard clinical routines – such phone calls and letters. Apart from these clinical routines, no extra effort will be made to ensure patients' adherence, to maximize generalizability of the treatment uptake.

Therapist competence and adherence

Therapists in both arms will be licensed clinical psychologists or psychologists under supervision employed at the two specialist OCD clinics. All sessions will be audiotaped, 20% of the recorded sessions will be randomly selected and rated by independent expert psychologists (two for each treatment) not otherwise involved in the project. Adherence to protocol will be rated using checklists developed specifically for the study and for each intervention. Therapist competence will be rated using the Cognitive Therapy Scale–Revised (CTS-R) (20).

For peer review only - http://bmjopen.bmj.com/site/about/guidelines.xhtml

Sample size calculation

A power calculation was conducted to determine the number of participants needed to test the primary hypothesis that B4DT will be non-inferior compared to gold standard CBT at the pre-specified margin of 4 points on the Y-BOCS. There are no specific guidelines on appropriate non-inferiority margins in research on OCD, however a common rule of thumb is half the placebo-controlled effect of gold-standard treatment versus control conditions (21). The chosen margin of 4 is smaller than half of the 10-point effect that has been demonstrated in ERP-trials using Y-BOCS (10). Importantly, from a clinical perspective, a 4-point effect is small enough to consider other factors than symptom reduction, e.g. patient preference, cost effectiveness, and adverse events. Last, this margin gives similar precision compared to previous non-inferiority studies on OCD (22).

Clinical outcome data from a recent randomized controlled trial (23) and regular clinical practice (B4DT) were obtained to simulate study data. The following parameters were used: A pre-treatment mean of 22.6 (SD = 3.78) for both arms, and a post-treatment mean of 12.9 (SD = 4.07) for the gold standard CBT arm. Statistical power was then assessed by simulating 10000 datasets assuming different true group differences. An ANCOVA was then conducted on each dataset to test for between-group differences at post-treatment accounting for pre-treatment scores. With 60 participants per arm, no difference in efficacy between the treatments, and 10% missing values, this test has >95% power to demonstrate non-inferiority (which was considered supported if the upper bound was less than 4). Details on this calculation, including analysis code, is provided in the online pre-registration (https://osf.io/w5bfp/).

Measurements

Supplemental table 1 presents the flow of the recruitment and treatment process and lists clinician-rated and self-rated assessments at the different time points.

The primary outcome measure is the blind assessor-rated Y-BOCS, administered at enrollment, at week 4, 7, and 15 (primary endpoint), and at the 7- and 16-month follow-ups (19). The Y-BOCS is a gold-standard instrument for measuring OCD-symptoms having shown excellent interrater reliability (intraclass coefficient of .98) and internal consistency (Chronbach's alpha of .89) (19) as well as good validity (24). Blinded assessors will undergo training prior to conducting clinician-rated assessment and practice on videos of OCD case examples. To be allowed to conduct assessments for this project, assessors will have to deviate no more than 1 point on single items, no more than 2 points on the obsession subscale or the compulsion subscale and no more than 4 points on the Y-BOCS total score compared to expert raters at the two clinics. The expert-rater scores will be obtained in the following manner: for a co-rating session, the experienced clinicians working at both clinics will rate the same case recording. The subgroup of the clinicians whose total score lies inside the range between the 25th and 75th

For peer review only - http://bmjopen.bmj.com/site/about/guidelines.xhtml

percentiles will be identified. The median values on each item in this subgroup will be considered the expert-rater values. Inter-rater reliability on the Y-BOCS will be reported.

Secondary clinician-administered outcome measures are the Clinical Global Impression – Severity and Improvement (CGI-S, CGI-I, blind-rated) (25), the Patient Exposure/Response Prevention Adherence Scale (PEAS) (26), the Structured Clinical Interview for DSM-5 for OCD and related disorders (SCID-5) (27) and the Mini International Neuropsychiatric Interview (28). SCID-5 and MINI will be administered at enrollment to confirm the OCD diagnosis and assess comorbidities.

Secondary self-rated outcome measures are the Obsessive-Compulsive Inventory–Revised (OCI-R) (29), self-rated Y-BOCS (30), Montgomery-Åsberg Depression Rating Scale–Self-Rated (MADRS-S) (31), Work and Social Adjustment Scale (WSAS) (32), Assessing Quality of Life 6 Dimensions (AQoL-6D) (33), Credibility/Expectancy Questionnaire (CEQ) (34), Working Alliance Inventory–Short Form Revised (WAI-SR) (35), the Treatment Inventory of Costs in Psychiatric Patients (TIC-P) resource use questionnaire (for economic evaluation) (36), and the Negative Effects Questionnaire (NEQ) (37). Participants will also rate their treatment preference measured on a 7-point Likert-type scale (pre-treatment and post-treatment). At the end of each treatment, the therapist will provide information on the number of sessions the patient attended as well as any extra time (additional phone calls, supervision, administrative tasks) spent due to the needs of the specific patient.

Treatment-response will be defined as a ≥35% reduction on the Y-BOCS and a Clinical Global Impression-Improvement (CGI-I) score of 1 (”very much improved”) or 2 (”much improved”). Remission will be defined as a score of ≤12 on the Y-BOCS and a Clinical Global Impression-Severity (CGI-S) rating of 1 (”normal, not at all ill”) or 2 (”borderline mentally ill”). Recovery will be defined as a sustained remission status at the two long-term follow-ups (38). Participants who complete less than 8 sessions of CBT or attend at less than 1.5 days of B4DT will be considered non-completers, unless the treatment is terminated by the therapist due to early recovery.

The participants who do not engage in treatment or drop out from the trial will be encouraged to take part in all outcome assessments, prioritizing the primary outcome measure at the primary end point if the burden of assessments needs to be reduced to increase compliance. In order to assess quality of the data inserted in the electronic case report forms (eCRF), all the eCRF:s will be compared to the source data for 10% of the participants be the quality monitor Karolinska Trial Alliance.

For peer review only - http://bmjopen.bmj.com/site/about/guidelines.xhtml

Safety and adverse events

Information on adverse events including suicidal ideation, and initiation of additional psychiatric care, will be collected at each assessment following inclusion, as well as through the regular contact between participants and therapists during treatment. The incidents will be handled according to the clinics' routines and described on a case-by-case basis. The NEQ with 32 items on incidents and negative effects will be used at the primary endpoint and follow-ups. Adverse events will be labeled serious if they are life-threatening, result in death or persistent/significant disability/incapacity, require hospitalization or its prolongation, or is considered medically significant by the principal investigator. All the personal data will be stored in locked archive rooms with personal access (analogue data), or on encrypted servers, requiring VPN-connection and/or two-step authentication (digital data) in accordance with Region Stockholm and Karolinska Institutet's data management guidelines.

Statistical analysis

Analysis code and a more detailed analysis plan were uploaded to Open Science Framework before any data had been collected (https://osf.io/w5bfp/, under Preregistration – Analysis).

Primary outcome

The primary non-inferiority hypothesis on the clinician-rated Y-BOCS will be evaluated using an ANCOVA of group differences at week 15 with pre-treatment score as covariate. It will be based on all treatment completers. This per-protocol approach is chosen to avoid potential problems with the intention-to-treat approach in a non-inferiority study design, which can bias the results towards the alternative hypothesis of non-inferiority (39). No data imputation will be conducted at this stage. A 95% confidence interval will be used to model the gold standard CBT vs. B4DT effect. Non-inferiority is supported if the upper bound is less than 4 (the non-inferiority margin). This testing procedure has the Type I error rate controlled at the 2.5% level for both comparisons.

As sensitivity analyses, the same ANCOVA model will be fitted with an intention-to-treat approach, including all randomized participants. Missing data will then be estimated using maximum likelihood after which the models are refitted. The aim of these steps is to address the potential influence of non-random missing data in the primary per protocol analysis.

Secondary outcomes

The superiority hypothesis (that B4DT is more effective than standard CBT) will be evaluated using the exact same model used for the main non-inferiority hypothesis, see Primary outcome,

For peer review only - http://bmjopen.bmj.com/site/about/guidelines.xhtml

and superiority will be considered supported if the upper bound of the 95% confidence interval is less than 0.

Speed of response, rates of response and remission, and dropout rates

For other secondary outcomes we will compute 95% confidence intervals of group differences using intent-to-treat approach without comparing the bounds to any predefined margin.Since we focus on pre-to-post-treatment comparisons, no data imputation methods will be therefore used as default (unless stated otherwise). However, if the proportion of missing data exceeds 10%, we will perform maximum likelihood imputations based on demographics and the scores from the pre-treatment assessment. To assess speed of response, responder status in the two treatment arms will be compared at week 4 and 7 using generalized linear model where 95% confidence intervals of the odds ratio of responder status will be reported. The same model will be used to compare responder and remitter status in the groups at week 15 to assess rates of response and remission. Proportions of dropouts in the two treatment groups will be compared using a two-sided two-proportions z-test.

Cost effectiveness

We will conduct a within-trial economic evaluation (40) whereby outcome and cost data will be compared at week 15. It will encompass three analyses: 1) a cost-effectiveness analysis using responder status as outcome; 2) a cost-effectiveness analysis using the remitter status as outcome; and 3) a cost-utility analysis using the outcome quality adjusted life years (QALYs) measured using the AQOL-6D (33).

Health related quality of life (HRQoL) will be collected using the AQOL-6D (33,41), a multiattribute utility instrument for use in economic evaluation. Data on resource use will be collected using an adapted version of the self-reported Treatment Inventory of Costs in Psychiatric Patients (TIC-P) questionnaire (36). Costs will be measured from three perspectives: a healthcare organization payer perspective (including intervention costs only), a healthcare system perspective (additionally including costs related to the use of medical resources, and medication) and a societal perspective (additionally including social care costs and productivity losses for the individual). Costs from each perspective will be analyzed in relation to clinical efficacy (responder status; cost-effectiveness analysis) and QALYs (cost utility analysis). Results will be presented as incremental cost-effectiveness ratios (ICER), as the ratio between the difference in costs and the difference in health outcomes ICERs will be put against values of willingness to pay for a QALY to determine cost-effectiveness.

Standard economic evaluation techniques will be used to explore uncertainty around the cost

For peer review only - http://bmjopen.bmj.com/site/about/guidelines.xhtml

and effect data, which will be represented on cost-effectiveness planes. The probability of cost-effectiveness according to different willingness to pay thresholds will be represented on cost effectiveness acceptability curves (42).

Adverse events

A regression analysis with treatment group as predictor will be used to model the total score from the NEQ at week 15. Additional semi-structured questions about severe adverse events will be reported in a frequency table.

Long-term maintenance of gains

At 7 and 16-month follow-ups we will test both the non-inferiority and superiority hypotheses, as well as assess the rates of response, remission and recovery, cost effectiveness and adverse events. For each outcome measure, we will use the same analysis method as at the primary endpoint described above. The generalized linear model used for rates of response and remission will be applied for rates of recovery.

Additional outcomes used for quality assessment

Therapist competence and adherence

Therapist competence (scored using the Cognitive Therapist Scale–Revised (CTS-R)) and protocol adherence will be presented using descriptive information (e.g., means and standard deviations), as well as inter-rater reliability between independent assessors.

Treatment preference, credibility, working alliance and patient adherence

Responses to the question about treatment preference will be presented descriptively at pre-treatment and week 15 (means and standard deviations). An exploratory analysis will evaluate whether the degree of agreement between pre-treatment preference and treatment received moderates the main Y-BOCS outcome.

Total score means with standard deviations for treatment credibility, working alliance, and patients' adherence will be presented for both treatments.

Patient and public involvement

Patient representatives have not been involved in the design of the current study. The Swedish OCD Foundation supports the study by providing information about the project to their

For peer review only - http://bmjopen.bmj.com/site/about/guidelines.xhtml

members and the public through their channels. Results from the study will be reported to the public and relevant patient organizations through open access publishing and lectures.

DISCUSSION

This study is the first trial to directly compare an intensive treatment for OCD, the Bergen 4-day treatment (B4DT) with gold standard individual CBT delivered over 14 weeks. The study is well powered to test whether B4DT is non-inferior to gold standard CBT in terms of symptom reduction with a 4-point non-inferiority margin. If the two treatments are comparable in terms of clinical efficacy, B4DT should be considered a candidate for implementation in regular care as the intensive format may be more suitable or preferable for some patients.

Limitations

First, the two recruitment sites in this study are located in different parts of Stockholm. This means that some participants might prefer to be randomized to the clinic closest to their home, which could pose a risk of selection bias. Second, this study does not have sufficient statistical power to robustly assess the secondary outcomes that will be interpreted as exploratory. Last, therapists and patients will be aware of their allocated treatment arm. However, extensive efforts have been made to improve blinding integrity.

ETHICS AND DISSEMINATION

Patient safety

The study has been approved by the Swedish Ethical Review Authority (EPM 2022-01713-01). All patients will receive either gold standard CBT or B4DT delivered by clinicians who are specialized in each of these forms of treatment delivery. Gold standard CBT is already the firsthand choice for this patient group, and its efficacy and safety have been demonstrated in several studies of high quality (10). While B4DT has not yet been evaluated in an RCT with an active control group, it is an intensive face-to-face treatment with an emphasis on ERP. Moreover, uncontrolled studies have suggested that the effect is similar, or even larger, compared to gold standard CBT (15–17). We therefore consider the additional risks associated with participating in the B4DT-arm to be small. All participants will be monitored closely during the study and additional treatment will be provided in case of severe deterioration.

Participants referred to the clinics by other health-care services who turn out not to be eligible will be offered alternative treatment in regular care. Excluded self-referred participants will be guided to adequate care.

For peer review only - http://bmjopen.bmj.com/site/about/guidelines.xhtml

Dissemination

We will adopt practices that are known to increase the trustworthiness of scientific research such as prospectively registering the study hypotheses and the statistical analysis plan, adhering to gold standard outcome reporting, controlling the risk of false positive results by interpreting the secondary analyses as exploratory, and sharing materials needed to evaluate our results (the analysis code, the protocol). A detailed description of the dataset will be saved in a searchable database, SND (43). The results of the trial will be communicated in open access scientific publications, at scientific conferences as well as meetings with representatives from health care system and patient organizations, and in public media.

CURRENT TRIAL STATUS

Inclusion started in November 2022 and is expected to end in June 2024. At the time of submission, 66 participants had been randomized. The last follow-up appointment is expected to take place in October 2025. Data analysis and reporting of results will begin when all data from the primary endpoint has been collected (October 2024).

For peer review only

For peer review only - http://bmjopen.bmj.com/site/about/guidelines.xhtml

ADDITIONAL INFORMATION

Study sponsor: Region Stockholm, Stockholm Sweden. Contact: managing director Lina Martinsson; lina.martinsson@regionstockholm.se; Psykiatri Sydväst, M 58 Huddinge sjukhusområde, 141 86 Stockholm; 08-123 800 00.

Role of funding sources

This study is partly funded by Helse Bergen, the hospital where B4DT was developed. Additional financial support for the study is provided by CIMED (FoUI-960456), Region Stockholm (ALF project RS2020-0731, ALF Postdoc FoUI-963608) and Stiftelsen Söderström-Königska Sjukhemmet (SLS-981976, SLS-980922).

The funders had no role in study design, data collection and analysis, decision to publish or preparation of the manuscript.

Author contributions

CR initiated the project and CR, VI and EI obtained funding for the trial. The study was designed in tight collaboration with DMC and EA. EI had the overall responsibility for the project's progression. RF and OF developed the pre-registered analyses plan and drafted this protocol, RF and EI finished it together. FS developed the cost-effectiveness analysis plan. SD, SA, and MS organized the work at the clinics. All authors collaborated on the proposal and methodology and critically reviewed the final version of this protocol.

Personal declaration of interest

Dr Flygare has received speaking fees from the Swedish OCD Association, Insight Events AB, and Kry International AB, as well as reimbursement for writing articles for Inside Practice Psychiatry; all outside the submitted work. Dr Ivanov has received speaking fees from the Swedish OCD Association outside the submitted work. Prof Mataix-Cols receives royalties for contributing articles to UpToDate, Inc., outside the submitted work. Dr Andersson and Ms Asplund receive royalties from Natur & Kultur for books, all outside the submitted work. Dr Rück receives royalties from Natur & Kultur, Studentlitteratur and Albert Bonniers Förlag and has received speaking fees from various sources, all outside the submitted work.

The rest of the authors have nothing to declare.

For peer review only - http://bmjopen.bmj.com/site/about/guidelines.xhtml

REFERENCES

1. American Psychiatric Association. Diagnostic and Statistical Manual of Mental Disorders (DSM-5®). American Psychiatric Pub; 2013. 1520 p.

2. Fontenelle IS, Fontenelle LF, Borges MC, Prazeres AM, Rangé BP, Mendlowicz MV, et al. Quality of life and symptom dimensions of patients with obsessive–compulsive disorder. Psychiatry Research. 2010 Sep;179(2):198–203.

3. Koran LM, Thienemann M, Davenport R. Quality of life for patients with obsessive-compulsive disorder. AJP. 1996 Jun;153(6):783–8.

4. Harvard Medical School. National Comorbidity Survey (NCSSC). Data Table 1: Lifetime prevalence DSM-IV/WMH-CIDI disorders by sex and cohort. [Internet]. 2007 [cited 2017 Aug 21]. Available from: https://www.hcp.med.harvard.edu/ncs/index.php

5. Fernández de la Cruz L, Lichtenstein P, Mataix-Cols D, D'Onofrio BM, Rück C, de la Cruz LF, et al. Suicide in obsessive–compulsive disorder: a population-based study of 36 788 Swedish patients. Mol Psychiatry. 2016 Jul 19;12(April):771–7.

6. Fernández de la Cruz L, Isomura K, Lichtenstein P, Rück C, Mataix-Cols D. Morbidity and mortality in obsessive-compulsive disorder: A narrative review. Neuroscience & Biobehavioral Reviews. 2022 May;136:104602.

7. Pérez-Vigil A, Fernández de la Cruz L, Brander G, Isomura K, Jangmo A, Feldman I, et al. Association of Obsessive-Compulsive Disorder With Objective Indicators of Educational Attainment: A Nationwide Register-Based Sibling Control Study. JAMA Psychiatry. 2018 Jan 1;75(1):47.

8. Pérez-Vigil A, Mittendorfer-Rutz E, Helgesson M, Cruz LF de la, Mataix-Cols D. Labour market marginalisation in obsessive–compulsive disorder: a nationwide register-based sibling control study. Psychological Medicine. 2018 Jun;1–10.

9. Rosa-Alcázar AI, Sánchez-Meca J, Gómez-Conesa A, Marín-Martínez F. Psychological treatment of obsessive-compulsive disorder: a meta-analysis. Clin Psychol Rev. 2008 Dec;28(8):1310–25.

10. Öst LG, Havnen A, Hansen B, Kvale G. Cognitive behavioral treatments of obsessive-compulsive disorder. A systematic review and meta-analysis of studies published 1993-2014. Clin Psychol Rev. 2015 Aug;40:156–69.

11. National Collaborating Centre for Mental Health (UK). Obsessive-Compulsive Disorder: Core Interventions in the Treatment of Obsessive-Compulsive Disorder and Body Dysmorphic Disorder [Internet]. Leicester (UK): British Psychological Society; 2006 [cited 2019 Mar 25]. (National Institute for Health and Clinical Excellence: Guidance). Available from: http://www.ncbi.nlm.nih.gov/books/NBK56458/

12. Foa EB, Yadin E, Lichner TK. Exposure and response (ritual) prevention for obsessive-compulsive disorder: therapist guide. 2nd ed. Oxford ; New York: Oxford University Press; 2012. 182 p. (Treatments that work).

13. Kvale G, Hansen B. Dissemination and Intensifying evidence-based treatment for OCD: Norway is in the lead. The Nordic Psychiatrist. 2014 Jan;3:14–5.

14. Havnen A, Hansen B, Öst LG, Kvale G. Concentrated ERP delivered in a group setting: An effectiveness study. Journal of Obsessive-Compulsive and Related Disorders. 2014 Oct 1;3(4):319–24.

15. Havnen A, Hansen B, Öst LG, Kvale G. Concentrated ERP Delivered in a Group Setting: A Replication Study. Behavioural and Cognitive Psychotherapy. 2017 Sep;45(5):530–6.

For peer review only - http://bmjopen.bmj.com/site/about/guidelines.xhtml

16. Hansen B, Kvale G, Hagen K, Havnen A, Öst LG. The Bergen 4-day treatment for OCD: four years follow-up of concentrated ERP in a clinical mental health setting. Cognitive Behaviour Therapy. 2018 Aug 8;0(0):1–17.

17. Hansen B, Hagen K, Öst LG, Solem S, Kvale G. The Bergen 4-day OCD treatment delivered in a group setting: 12-month follow-up. Front Psychol [Internet]. 2018 [cited 2018 Apr 21];9. Available from: https://www.frontiersin.org/articles/10.3389/fpsyg.2018.00639/abstract

18. Launes G, Hagen K, Sunde T, Öst LG, Klovning I, Laukvik IL, et al. A Randomized Controlled Trial of Concentrated ERP, Self-Help and Waiting List for Obsessive- Compulsive Disorder: The Bergen 4-Day Treatment. Front Psychol [Internet]. 2019 [cited 2019 Nov 19];10. Available from: https://www.frontiersin.org/articles/10.3389/fpsyg.2019.02500/full?utm_source=F-NTF&utm_medium=EMLX&utm_campaign=PRD_FEOPS_20170000_ARTICLE

19. Goodman WK, Price LH, Rasmussen SA, Mazure C, Delgado P, Heninger GR, et al. The Yale-Brown Obsessive Compulsive Scale. I. Development, Use, and Reliability. Arch Gen Psychiatry. 1989 Nov;46(11):1012–6.

20. Blackburn IM, James IA, Milne DL, Baker C, Standart S, Garland A, et al. The Revised Cognitive Therapy Scale (CTS-R): Psychometric Properties. Behav Cogn Psychother. 2001 Oct;29(4):431–46.

21. Committee for medicinal products for human use (CHMP) guideline on the choice of the non-inferiority margin. Statist Med. 2006 May 30;25(10):1628–38.

22. Lovell K, Cox D, Haddock G, Jones C, Raines D, Garvey R, et al. Telephone administered cognitive behaviour therapy for treatment of obsessive compulsive disorder: randomised controlled non-inferiority trial. BMJ. 2006 Oct 28;333(7574):883.

23. Lundström L, Flygare O, Andersson E, Enander J, Bottai M, Ivanov VZ, et al. Effect of Internet-Based vs Face-to-Face Cognitive Behavioral Therapy for Adults With Obsessive-Compulsive Disorder: A Randomized Clinical Trial. JAMA Network Open. 2022 Mar 14;5(3):e221967.

24. Goodman WK. The Yale-Brown Obsessive Compulsive Scale: II. Validity. Arch Gen Psychiatry. 1989 Nov 1;46(11):1012.

25. Guy W (Editor). ECDEU assessment manual for psychopharmacology. Rockville, MD: US Department of Health, Education, and Welfare, Public Health Service, Alcohol, Drug Abuse, and Mental Health Administration; 1976.

26. Simpson HB, Maher M, Page JR, Gibbons CJ, Franklin ME, Foa EB. Development of a Patient Adherence Scale for Exposure and Response Prevention Therapy. Behavior Therapy. 2010 Mar;41(1):30–7.

27. First MB, Williams JBW, Karg RS, Spitzer RL. Structured clinical interview for DSM-5 - Research version. Arlington, VA: American Psychiatric Association; 2015.

28. Sheehan DV, Lecrubier Y, Sheehan KH, Amorim P, Janavs J, Weiller E, et al. The Mini-International Neuropsychiatric Interview (M.I.N.I.): the development and validation of a structured diagnostic psychiatric interview for DSM-IV and ICD-10. J Clin Psychiatry. 1998;59 Suppl 20:22-33;quiz 34-57.

29. Foa EB, Huppert JD, Leiberg S, Langner R, Kichic R, Hajcak G, et al. The Obsessive-Compulsive Inventory: Development and validation of a short version. Psychological Assessment. 2002;14(4):485–96.

30. Steketee G, Frost R, Bogart K. The Yale-Brown Obsessive Compulsive Scale: Interview versus self-report. Behaviour Research and Therapy. 1996 Aug;34(8):675–84.

For peer review only

For peer review only - http://bmjopen.bmj.com/site/about/guidelines.xhtml

31. Svanborg P, Åsberg M. A comparison between the Beck Depression Inventory (BDI) and the self-rating version of the Montgomery Åsberg Depression Rating Scale (MADRS). Journal of Affective Disorders. 2001 May;64(2–3):203–16.

32. Mundt JC, Marks IM, Shear MK, Greist JM. The Work and Social Adjustment Scale: a simple measure of impairment in functioning. Br J Psychiatry. 2002 May;180(5):461–4.

33. Richardson JR, Peacock SJ, Hawthorne G, Iezzi A, Elsworth G, Day NA. Construction of the descriptive system for the assessment of quality of life AQoL-6D utility instrument. Health Qual Life Outcomes. 2012;10(1):38.

34. Devilly GJ, Borkovec TD. Psychometric properties of the credibility/expectancy questionnaire. Journal of Behavior Therapy and Experimental Psychiatry. 2000 Jun;31(2):73–86.

35. Hatcher RL, Gillaspy JA. Development and validation of a revised short version of the working alliance inventory. Psychotherapy Research. 2006 Jan 1;16(1):12–25.

36. Timman R, Bouwmans C, Busschbach JJV, Hakkaart-van Roijen L. Development of the Treatment Inventory of Costs in Psychiatric Patients: TIC-P Mini and Midi. Value in Health. 2015 Dec;18(8):994–9.

37. Rozental A, Kottorp A, Forsström D, Månsson K, Boettcher J, Andersson G, et al. The Negative Effects Questionnaire: psychometric properties of an instrument for assessing negative effects in psychological treatments. 2019;47(5):559–72.

38. Mataix-Cols D, Fernández de la Cruz L, Nordsletten AE, Lenhard F, Isomura K, Simpson HB. Towards an international expert consensus for defining treatment response, remission, recovery and relapse in obsessive-compulsive disorder. World Psychiatry. 2016 Feb;15(1):80–1.

39. Food and Drug Administration. Non-inferiority clinical trials to establish effectiveness: guidance for industry [Internet]. US: Department of Health and Human Services; 2016 [cited 2023 May 11]. Available from: https://www.fda.gov/media/78504/download

40. Drummond M. Methods for the economic evaluation of health care programmes. Fourth edition. Oxford, United Kingdom ; New York, NY, USA: Oxford University Press; 2015. 445 p. (Oxford medical publications).

41. Maxwell A, Özmen M, Iezzi A, Richardson J. Deriving population norms for the AQoL-6D and AQoL-8D multi-attribute utility instruments from web-based data. Qual Life Res. 2016 Dec;25(12):3209–19.

42. Fenwick E, Byford S. A guide to cost-effectiveness acceptability curves. Br J Psychiatry. 2005 Aug;187(2):106–8.

43. Munafò MR, Nosek BA, Bishop DVM, Button KS, Chambers CD, Percie du Sert N, et al. A manifesto for reproducible science. Nat Hum Behav. 2017 Jan 10;1(1):0021.

For peer review only

For peer review only - http://bmjopen.bmj.com/site/about/guidelines.xhtml

FIGURE LEGENDS

Figure 1. CONSORT-flow diagram. Abbreviations: B4DT, Bergen 4-day treatment; CBT, cognitive behavior therapy.

For peer review only

For peer review only - http://bmjopen.bmj.com/site/about/guidelines.xhtml

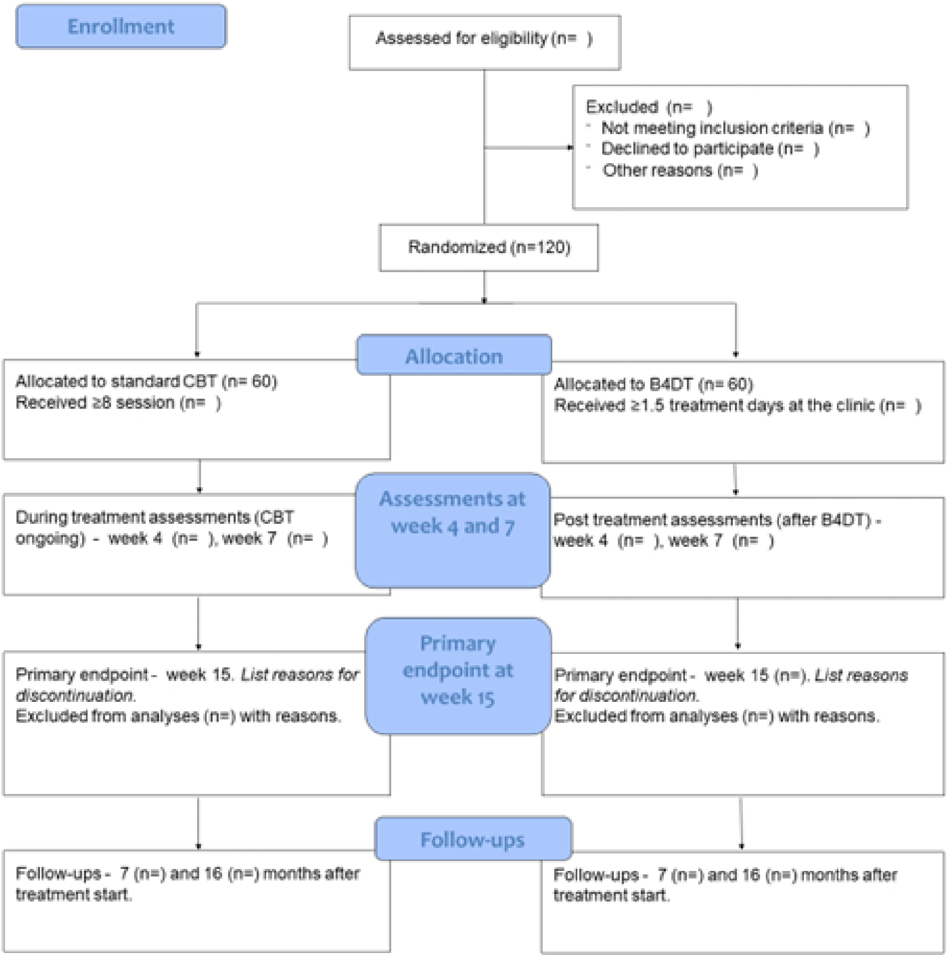

Figure 1. CONSORT-flow diagram. Abbreviations: B4DT, Bergen 4-day treatment; CBT, cognitive behavior therapy.

348x350mm (38 x 38 DPI)

Study protocol for a single-blind, parallel-group, randomized, controlled non-inferiority trial of four-day intensive versus standard cognitive behavioral therapy for adults with obsessive-compulsive disorder

Supplemental table 1. Overview of the recruitment, treatments and assessment points.

	Enrollment	Allocation	Pre-treatment	Week 1-2	Week 4	Week 5-6	Week 7	Week 8-14	Week 15 (primary end-point)	7-month follow-up	16-month follow-up
Enrollment:											
Eligibility screen	X										
Informed consent	X										
Allocation		X									
Treatment											
Gold-standard CBT				X	X	X	X	X			
B4DT				X							
Clinician-rated instruments											
SCID-5 (OCD)	X										
MINI	X										
Y-BOCS	X				X		X		X	X	X
CGI-S	X				X		X		X	X	X
CGI-I					X		X		X	X	X
PEAS*					X				X		
Self-rated instruments											
YBOCS-SR			X						X	X	X
OCI-R			X						X	X	X
MADRS-S			X						X	X	X
WSAS			X						X	X	X
AQoL-6D			X						X	X	X
TIC-P			X						X	X	X
NEQ									X	X	X

For peer review only

Other adverse events					X		X		X	X	X
CEQ**				X							
WAI-SR**				X							
Treatment preference			X						X		

Abbreviations: AQoL-6D, Assessing Quality of Life 6 Dimensions; CEQ, Credibility/Expectancy Questionnaire; CGI-I, Clinical Global Impression–Improvement; CGI-S, Clinical Global Impression–Severity; MADRS-S; Montgomery-Åsberg Depression Rating Scale–Self-Rated; MINI, Mini International Neuropsychiatric Interview; NEQ, Negative Effects Questionnaire; OCI-R, Obsessive-Compulsive Inventory–Revised; PEAS, Patient Exposure/Response Prevention Adherence Scale; SCID-5, Structured Clinical Interview for DSM-5 Disorders; TIC-P, Treatment Inventory of Costs in Psychiatric Patients; WAI-SR, Working Alliance Inventory–Short Form Revised; WSAS, Work and Social Adjustment Scale; Y-BOCS, Yale-Brown Obsessive Compulsive Scale; Y-BOCS-SR, Yale-Brown Obsessive Compulsive Scale–Self-Rated; Other adverse events, semi-structured questions about potential severe adverse events and psychiatric care outside the study

* PEAS rated once at the last day of B4DT treatment in the B4DT-arm

** CEQ and WAI-SR rated by participants at the last day of B4DT treatment and week 2 in individual CBT

For peer review only

STANDARD PROTOCOL ITEMS: RECOMMENDATIONS FOR INTERVENTIONAL TRIALS

SPIRIT 2013 Checklist: Recommended items to address in a clinical trial protocol and related documents*

Section/item	Item No	Description	Addressed on page number
**Administrative information**			
Title	1	Descriptive title identifying the study design, population, interventions, and, if applicable, trial acronym	1
Trial registration	2a	Trial identifier and registry name. If not yet registered, name of intended registry	2
	2b	All items from the World Health Organization Trial Registration Data Set	Appendix 2
Protocol version	3	Date and version identifier	1
Funding	4	Sources and types of financial, material, and other support	16
Roles and responsibilities	5a	Names, affiliations, and roles of protocol contributors	1
	5b	Name and contact information for the trial sponsor	16
	5c	Role of study sponsor and funders, if any, in study design; collection, management, analysis, and interpretation of data; writing of the report; and the decision to submit the report for publication, including whether they will have ultimate authority over any of these activities	16
	5d	Composition, roles, and responsibilities of the coordinating centre, steering committee, endpoint adjudication committee, data management team, and other individuals or groups overseeing the trial, if applicable (see Item 21a for data monitoring committee)	4, 6, 10

For peer review only - http://bmjopen.bmj.com/site/about/guidelines.xhtml

Introduction

Background and rationale	6a	Description of research question and justification for undertaking the trial, including summary of relevant studies (published and unpublished) examining benefits and harms for each intervention	3
	6b	Explanation for choice of comparators	3
Objectives	7	Specific objectives or hypotheses	4
Trial design	8	Description of trial design including type of trial (eg, parallel group, crossover, factorial, single group), allocation ratio, and framework (eg, superiority, equivalence, noninferiority, exploratory)	4

Methods: Participants, interventions, and outcomes

Study setting	9	Description of study settings (eg, community clinic, academic hospital) and list of countries where data will be collected. Reference to where list of study sites can be obtained	4
Eligibility criteria	10	Inclusion and exclusion criteria for participants. If applicable, eligibility criteria for study centres and individuals who will perform the interventions (eg, surgeons, psychotherapists)	5-6, 7
Interventions	11a	Interventions for each group with sufficient detail to allow replication, including how and when they will be administered	6-7, 9-10
	11b	Criteria for discontinuing or modifying allocated interventions for a given trial participant (eg, drug dose change in response to harms, participant request, or improving/worsening disease)	11
	11c	Strategies to improve adherence to intervention protocols, and any procedures for monitoring adherence (eg, drug tablet return, laboratory tests)	7, 10
	11d	Relevant concomitant care and interventions that are permitted or prohibited during the trial	5
Outcomes	12	Primary, secondary, and other outcomes, including the specific measurement variable (eg, systolic blood pressure), analysis metric (eg, change from baseline, final value, time to event), method of aggregation (eg, median, proportion), and time point for each outcome. Explanation of the clinical relevance of chosen efficacy and harm outcomes is strongly recommended	8-11
Participant timeline	13	Time schedule of enrolment, interventions (including any run-ins and washouts), assessments, and visits for participants. A schematic diagram is highly recommended (see Figure)	9-10

For peer review only - http://bmjopen.bmj.com/site/about/guidelines.xhtml

Sample size	14	Estimated number of participants needed to achieve study objectives and how it was determined, including clinical and statistical assumptions supporting any sample size calculations	8
Recruitment	15	Strategies for achieving adequate participant enrolment to reach target sample size	5

Methods: Assignment of interventions (for controlled trials)

Allocation:

Sequence generation	16a	Method of generating the allocation sequence (eg, computer-generated random numbers), and list of any factors for stratification. To reduce predictability of a random sequence, details of any planned restriction (eg, blocking) should be provided in a separate document that is unavailable to those who enrol participants or assign interventions	6
Allocation concealment mechanism	16b	Mechanism of implementing the allocation sequence (eg, central telephone; sequentially numbered, opaque, sealed envelopes), describing any steps to conceal the sequence until interventions are assigned	6
Implementation	16c	Who will generate the allocation sequence, who will enrol participants, and who will assign participants to interventions	6
Blinding (masking)	17a	Who will be blinded after assignment to interventions (eg, trial participants, care providers, outcome assessors, data analysts), and how	6
	17b	If blinded, circumstances under which unblinding is permissible, and procedure for revealing a participant's allocated intervention during the trial	NA

Methods: Data collection, management, and analysis

Data collection methods	18a	Plans for assessment and collection of outcome, baseline, and other trial data, including any related processes to promote data quality (eg, duplicate measurements, training of assessors) and a description of study instruments (eg, questionnaires, laboratory tests) along with their reliability and validity, if known. Reference to where data collection forms can be found, if not in the protocol	10-11
	18b	Plans to promote participant retention and complete follow-up, including list of any outcome data to be collected for participants who discontinue or deviate from intervention protocols	10

For peer review only - http://bmjopen.bmj.com/site/about/guidelines.xhtml

Data management	19	Plans for data entry, coding, security, and storage, including any related processes to promote data quality (eg, double data entry; range checks for data values). Reference to where details of data management procedures can be found, if not in the protocol	10
Statistical methods	20a	Statistical methods for analysing primary and secondary outcomes. Reference to where other details of the statistical analysis plan can be found, if not in the protocol	11-14
	20b	Methods for any additional analyses (eg, subgroup and adjusted analyses)	11-14
	20c	Definition of analysis population relating to protocol non-adherence (eg, as randomised analysis), and any statistical methods to handle missing data (eg, multiple imputation)	11-14
**Methods: Monitoring**			
Data monitoring	21a	Composition of data monitoring committee (DMC); summary of its role and reporting structure; statement of whether it is independent from the sponsor and competing interests; and reference to where further details about its charter can be found, if not in the protocol. Alternatively, an explanation of why a DMC is not needed	4, 6, 10
	21b	Description of any interim analyses and stopping guidelines, including who will have access to these interim results and make the final decision to terminate the trial	NA
Harms	22	Plans for collecting, assessing, reporting, and managing solicited and spontaneously reported adverse events and other unintended effects of trial interventions or trial conduct	11
Auditing	23	Frequency and procedures for auditing trial conduct, if any, and whether the process will be independent from investigators and the sponsor	4, 6, 10
**Ethics and dissemination**			
Research ethics approval	24	Plans for seeking research ethics committee/institutional review board (REC/IRB) approval	4-5
Protocol amendments	25	Plans for communicating important protocol modifications (eg, changes to eligibility criteria, outcomes, analyses) to relevant parties (eg, investigators, REC/IRBs, trial participants, trial registries, journals, regulators)	5

For peer review only - http://bmjopen.bmj.com/site/about/guidelines.xhtml

Consent or assent	26a	Who will obtain informed consent or assent from potential trial participants or authorised surrogates, and how (see Item 32)	5
	26b	Additional consent provisions for collection and use of participant data and biological specimens in ancillary studies, if applicable	NA
Confidentiality	27	How personal information about potential and enrolled participants will be collected, shared, and maintained in order to protect confidentiality before, during, and after the trial	11
Declaration of interests	28	Financial and other competing interests for principal investigators for the overall trial and each study site	NA
Access to data	29	Statement of who will have access to the final trial dataset, and disclosure of contractual agreements that limit such access for investigators	15
Ancillary and post-trial care	30	Provisions, if any, for ancillary and post-trial care, and for compensation to those who suffer harm from trial participation	11
Dissemination policy	31a	Plans for investigators and sponsor to communicate trial results to participants, healthcare professionals, the public, and other relevant groups (eg, via publication, reporting in results databases, or other data sharing arrangements), including any publication restrictions	15
	31b	Authorship eligibility guidelines and any intended use of professional writers	15
	31c	Plans, if any, for granting public access to the full protocol, participant-level dataset, and statistical code	2, 11
**Appendices**			
Informed consent materials	32	Model consent form and other related documentation given to participants and authorised surrogates	Appendix 1
Biological specimens	33	Plans for collection, laboratory evaluation, and storage of biological specimens for genetic or molecular analysis in the current trial and for future use in ancillary studies, if applicable	NA

*It is strongly recommended that this checklist be read in conjunction with the SPIRIT 2013 Explanation & Elaboration for important clarification on the items. Amendments to the protocol should be tracked and dated. The SPIRIT checklist is copyrighted by the SPIRIT Group under the Creative Commons "Attribution-NonCommercial-NoDerivs 3.0 Unported" license.

For peer review only - http://bmjopen.bmj.com/site/about/guidelines.xhtml
